# Approaching the Complete Basis Set Limit for Spin-State Energetics of Mononuclear First-Row Transition Metal Complexes

**DOI:** 10.1021/acs.jctc.4c00092

**Published:** 2024-04-04

**Authors:** Gabriela Drabik, Mariusz Radoń

**Affiliations:** † Jagiellonian University, Doctoral School of Exact and Natural Sciences, Łojasiewicza 11, 30-348 Kraków, Poland; ‡ Jagiellonian University, Faculty of Chemistry, Gronostajowa 2, 30-387, Kraków Poland

## Abstract

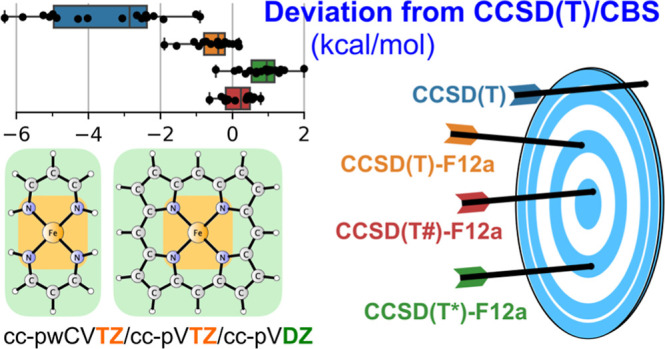

Convergence to the complete basis set (CBS) limit is analyzed for the problem of spin-state energetics in mononuclear first-row transition metal (TM) complexes by taking under scrutiny a benchmark set of 18 energy differences between spin states for 13 chemically diverse TM complexes. The performance of conventional CCSD(T) and explicitly correlated CCSD(T)-F12a/b calculations in approaching the CCSD(T)/CBS limits is systematically studied. An economic computational protocol is developed based on the CCSD-F12a approximation and (here proposed) modified scaling of the perturbative triples term (T#). This computational protocol recovers the relative spin-state energetics of the benchmark set in excellent agreement with the reference CCSD(T)/CBS limits (mean absolute deviation of 0.4, mean signed deviation of 0.2, and maximum deviation of 0.8 kcal/mol) and enables performing canonical CCSD(T) calculations for mononuclear TM complexes sized up to ca. 50 atoms, which is illustrated by application to heme-related metalloporphyrins. Furthermore, a good transferability of the basis set incompleteness error (BSIE) is demonstrated for spin-state energetics computed using CCSD(T) and other wave function methods (MP2, CASPT2, CASPT2/CC, NEVPT2, and MRCI + Q), which justifies efficient focal-point approximations and simplifies the construction of multimethod benchmark studies.

## 1 Introduction

Spin states resulting from different distributions of electrons on the metal d orbitals in transition metal (TM) complexes are highly relevant in inorganic chemistry, biochemistry, catalysis, and materials science.^1−4^ Nevertheless, reliable computation of spin-state energetics (i.e., the energy differences between alternative spin states; also termed spin-state splittings) poses a formidable challenge for computational chemistry methods.^5−9^ During the past decade, there have been numerous attempts to compute the spin-state energetics accurately using density functional theory (DFT)^10−15^ or wave function theory (WFT) methods.^12,16−27^ Despite the progress made, it also has been realized that the problem is very challenging due to a complicated interplay of dynamic and nondynamic correlation effects in TM complexes, so that divergent results are often reported even at high theory levels.^9^ Partly for this reason, but also due to the scarcity of definite benchmarks, practical computational protocols for predicting TM spin-state energetics unarguably within the chemical accuracy (i.e., ±1 kcal/mol) from the experimental values are still to be sought.

As generally observed in quantum chemistry, the problem of accurate energy computation has two dimensions: the first one is the quality of the electronic structure method used to approximate the full configuration interaction (FCI) limit and the second one is the level of completeness for the basis set in which the molecular orbitals are expanded. It is the second dimension that is in focus of the present article. The quality of basis set is particularly relevant for conventional WFT methods due to their painfully slow convergence toward the complete basis set (CBS) limit, which is caused by the difficulty of reproducing the two-electron correlation cusps in the wave function by the products of one-electron functions.^28^ The result computed using a given method with an incomplete basis set differs from the method’s CBS limit by the quantity known as basis set incompleteness error (BSIE). The BSIE should be clearly distinguished from the method’s intrinsic error (IE), i.e., the difference between the method’s CBS limit and the exact solution of the Schrödinger equation.^29^ This distinction is particularly important in benchmark studies based on the comparison of computed results with experimental references: the observed discrepancy is a measure of the method’s IE only if the BSIE is substantially smaller. In practice, large remaining BSIEs will spoil the accuracy of computed results even for methods having small IEs. Because increasing the basis set size to make the BSIE negligibly small by brute force is impractical in larger molecules, it becomes a pressing problem to develop computational protocols for approaching the CBS limit accurately and efficiently.

The CBS limit can be approached by extrapolating the results obtained with two (or more) successively increasing basis sets. This approach hinges on the usage of an extrapolation formula (one of many approximate formulas that were proposed in the literature; see, e.g., the review provided by Feller, Peterson, and Hill in ref (30)) and the availability of systematically convergent basis sets (of which the most common ones are Dunning-style correlation-consistent (cc) basis sets,^31,32^ but other families of basis sets can be used as well^33^). Alternatively, the convergence of correlation energy to the CBS limit can be accelerated by making a wave function explicitly dependent on the interelectronic distance, which is exploited in modern explicitly correlated methods,^34^ for example, MP2-F12 or one of several approximate variants of the CCSD-F12 method.^35−37^ The F12 methods and basis set extrapolation techniques can be combined.^38^

The effectiveness of basis set extrapolations and F12 methods has been studied extensively (e.g., refs (30, 33, 38–44)), but mainly for molecules composed of main-group elements, whereas the number of similar systematic studies for TM-containing molecules is considerably smaller.^45−47^ The applications of F12 methods to TM systems have been for long hampered by the lack of F12-specialized basis sets for TM atoms, only recently developed by Martin with co-workers,^48^ although standard cc basis sets can be used too, as was demonstrated by Peterson with co-workers^47^ in their extensive study of atomization energies for small molecules containing TMs, by Harvey with co-workers,^49^ who pioneered F12 applications to realistic open-shell TM complexes, and by other groups,^25a,50^ including us.^17,26,27^ However, systematic basis set convergence studies are scarce for TM complexes. Most of the systematic studies available in the literature are focused on atomization energies of small molecules or intermolecular interactions of closed-shell molecules, neither of which is directly comparable to the presently considered problem of spin-state energetics: TM complexes are usually large, open-shell molecules, and their alternative spin states are stable energy minima with qualitatively identical patterns of chemical bonds but different numbers of unpaired electrons on the TM center. This local character of spin transitions supports the occurrence of error cancellations in energy contributions from the molecule’s regions lying far from the TM center. On the other hand, the spin transition affects the metal–ligand covalency and nondynamic correlation effects,^51−53^ making it very challenging in practice to accurately recover the differential correlation energy and converge it to the CBS limit.^9,16,22,25^ Moreover, the large molecular size of some practically interesting TM complexes—for example, spin-crossover (SCO) complexes^4^ and metalloporphyrins^17,54^—renders the calculations with extended basis sets very time-consuming or simply intractable. Thus, although excellent, highly accurate thermochemical protocols are available for small molecules containing TMs,^47,55−57^ it may be impractical (or simply impossible) to apply such methods in the computational studies of spin-state energetics for larger complexes. Therefore, an important goal of the present work is to develop computational protocols tailored to the spin-state energetics problem and being applicable to larger complexes. Our aim is not to achieve subchemical accuracy (like in many, impressively accurate thermochemical studies of small molecules) but rather to reduce the BSIE in the computed energy differences to below 1 kcal/mol in a systematic and computationally efficient way.

Most of the calculations in this article are performed with the well-known coupled cluster (CC) singles, doubles, and noniterative triples method, abbreviated CCSD(T).^58,59^ Our focus on this method is motivated by two factors. First, CCSD(T) is the “gold standard” approach in many areas of computational chemistry, and the available data suggest that it is also reasonably accurate for TM-containing molecules,^46,47,55,56,60,61^ including spin-state energetics of TM complexes.^17,22,26,27^ Even if the CCSD(T)’s accuracy is not always sufficient in all applications (notably, some authors expressed opinions^12,25a^ that the method has chemically significant IEs for spin-state energetics of some TM complexes), the CCSD(T) energy well converged to the CBS limit is required in the majority of accurate thermochemical protocols to eventually supplement it with higher-level corrections. Second, CCSD(T) is computationally very expensive: it has unfavorable scaling of the computation time and resources needed with the number of correlated electrons and the number of basis functions. This makes it a practical challenge to perform CCSD(T) calculations for larger molecules while keeping the resulting BSIE acceptably small. We note that although there exist promising local correlation approaches for CCSD(T), such as pair-natural orbital (PNO)^62^ or domain-based local PNO (DLPNO)^63^ approximations, our focus will be on the canonical CCSD(T) method with the aim of clearly separating the issue of basis set convergence from the truncation error of a local correlation approach. However, a limited comparison of the present canonical CCSD(T) results and the DLPNO–CCSD(T) results from the literature^64^ will be given for one studied complex.

In this article, we study in detail the basis set convergence of conventional and explicitly correlated CCSD(T) spin-state energetics for the new benchmark set of 13 small, mononuclear first-row TM complexes. The set includes Fe^II^, Fe^III^, Co^II^, and Mn^II^ complexes in different coordination architectures in order to represent the diversity of TMs and bonding types in bioinorganic and organometallic chemistry. Some of the complexes are simplified models of SCO complexes or metalloporphyrins, but all of them are still small enough to enable computationally expensive extrapolations or F12 approaches already tested in the literature. The number and diversity of presently studied systems is considerably larger than in any of the previous similar studies in which the basis set convergence of CCSD(T) spin-state energetics was investigated,^16,22,25^ allowing us to obtain here the set of data with greater statistical relevance. Based on the detailed analysis of the BSIE as a function of basis set, we will construct and validate an economic computational protocol tailored to approach the CCSD(T)/CBS accuracy for TM spin-state energetics at a largely reduced computational cost, taking advantage of the explicit correlation treatment. We will illustrate the superior performance of this computational protocol compared with that of alternative approaches as well as its applicability to study spin-state energetics of much larger TM complexes, such as metalloporphyrin models.

In addition to the regular CCSD(T) based on HF orbitals, we also consider a variant based on Kohn–Sham (KS) reference orbitals [KS-CCSD(T)] and a number of other WFT methods often applied to TM complexes:^7,19,20,26,65^ CASPT2, NEVPT2 (two variants of the second-order perturbation theory based on complete active space self-consistent field, CASSCF), the composite CASPT2/CC method of Phung et al.,^19^ and MRCI + Q (variational multireference configuration interaction with singles and doubles, and approximate size-consistency correction). Our main goal will be to learn about the transferability of the BSIEs in the spin-state energetics computed using different WFT methods. The concept of BSIE transferability will bring insight into the accuracy of focal-point approaches for computing TM spin-state energetics, but is also relevant for the construction of benchmark studies in which the results of different methods are compared with experimentally derived reference data.^9,26,27^

## 2 Methods and Models

### 2.1 Description of the Benchmark Set

**Figure 1:**
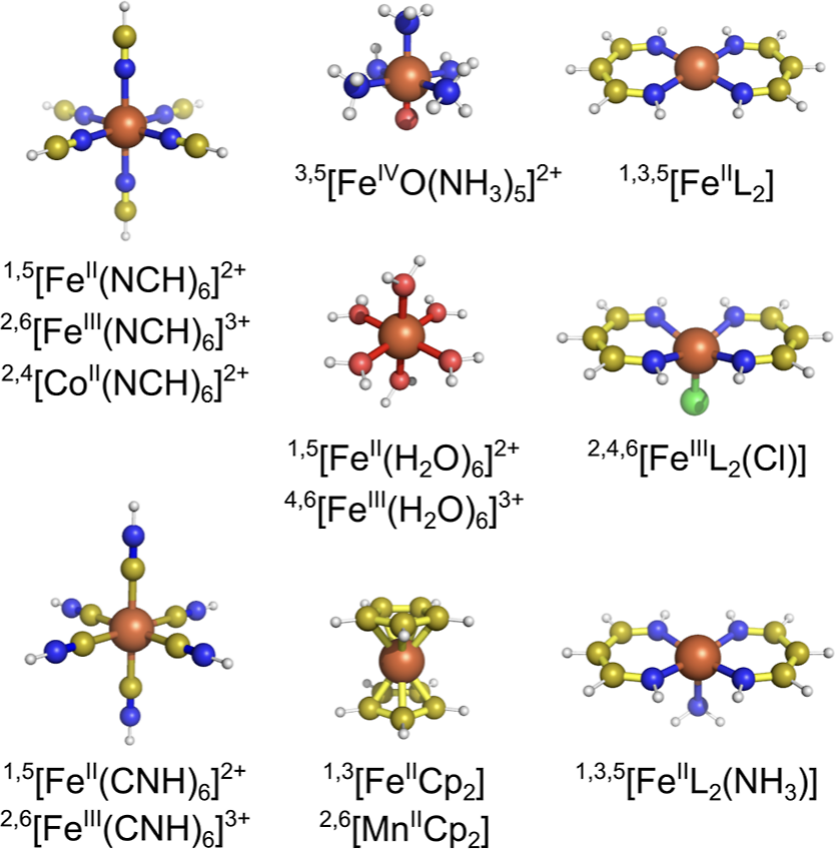
Structures of 13 small TM complexes making up the benchmark set (L^–^ = N_2_C_3_H_5_^–^, Cp^–^ = C_5_H_5_^–^). Superscripts give multiplicities of the investigated spin states.

The basis set convergence of spin-state energetics has been studied for the benchmark set of 18 energy differences for 13 mononuclear first-row TM complexes, whose structures are shown in Figure 1.

Table 1 describes the investigated energy differences, state symmetries, and the source of molecular geometries (in most cases, taken from the literature).

**Table 1:** Energy Differences in the Benchmark Set

item	spin states		*sym*^a^	geom^b^
^1,5^[Fe(NCH)_6_]^2+^	^1^*A*_*g*_	^5^*B*_1*g*_	*D*_2*h*_	ref (16)
^2,6^[Fe(NCH)_6_]^3+^	^2^*B*_1*g*_	^6^*A*_*g*_	*D*_2*h*_	this work^e^
^2,4^[Co(NCH)_6_]^2+^	^2^*A*_*g*_	^4^*B*_1*g*_	*D*_2*h*_	ref (16)
^1,5^[Fe(CNH)_6_]^2+^	^1^*A*_*g*_	^5^*B*_1*g*_	*D*_2*h*_	ref (24)
^2,6^[Fe(CNH)_6_]^3+^	^2^*B*_1*g*_	^6^*A*_*g*_	*D*_2*h*_	ref (24)
^3,5^[FeO(NH_3_)_5_]^2+^	^3^*A*″	^5^*A*′	*C*_*s*_	ref (20)
^1,5^[Fe(H_2_O)_6_]^2+^	^1^*A*_*g*_	^5^*B*_1*g*_	*D*_2*h*_	ref (24)
^4,6^[Fe(H_2_O)_6_]^3+^ (ve)^c^	^4^*B*_3*g*_	^6^*A*_*g*_	*D*_2*h*_	ref (66)
^1,3^[FeCp_2_]	^1^*A*_1_	^3^*B*_1_	*C*_2*v*_	ref (27)^e^
^1,3^[FeCp_2_] (ve)^d^	^1^*A*_1_	^3^*B*_2_		
^2,6^[MnCp_2_]	^2^*A*_1_	^6^*A*_*g*_	*C*_2*v*_/*C*_2*h*_	ref (27)^e^
^1,5^[FeL_2_]	^1^*A*_*g*_	^5^*A*_*g*_	*D*_2*h*_	ref (17)^f^
^3,5^[FeL_2_] (1)	^3^*B*_1*g*_	^5^*A*_*g*_		
^3,5^[FeL_2_] (2)	^3^*B*_3*g*_	^5^*A*_*g*_		
^2,6^[FeL_2_(Cl)]	^2^*B*_2_	^6^*A*_1_	*C*_2*v*_	ref (17)^g^
^4,6^[FeL_2_(Cl)]	^4^*A*_2_	^6^*A*_1_		
^1,5^[FeL_2_(NH_3_)]	^1^*A*′	^5^*A*′	*C*_*s*_	ref (17)^g^
^3,5^[FeL_2_(NH_3_)]	^3^*A*″	^5^*A*′		

a Symmetry point group used in the calculations (the true symmetry of some complexes is higher).

b Source of molecular geometries.

c Vertical energy (sextet geometry).

d Vertical energy (singlet geometry).

e PBE0/def2-TZVP.

f B3LYP/def2-TZVP.

g BP86/def2-TZVP.

The orbital occupancies and Cartesian coordinates can be found in Supporting Information. Altogether, there are nine energy differences with Δ*S* = 2 and 9 with Δ*S* = 1 (where *S* is the total spin quantum number). Most of them are adiabatic energies (i.e., each spin state is calculated in its own energy minimum) with the exception of two vertical energies, identified with the suffix “(ve)”: the sextet–quartet splitting of  and the triplet–singlet splitting of FeCp_2_ (for the latter metallocene molecule, both adiabatic and vertical energies are considered as they differ considerably^9,27^). Note that for FeL_2_, FeL_2_(NH_3_), and FeL_2_(Cl), which are simplistic models of metalloporphyrins,^17^ we consider the energy differences involving not only their low-spin (LS) and high-spin (HS) states but also the intermediate-spin (IS) state. Furthermore, for FeL_2_, the model of Fe^II^–porphyrin (FeP), we consider two IS states, corresponding to the nondegenerate and degenerate triplet states in FeP.^17^ The LS state considered for FeL_2_ is the closed-shell singlet, as in ref (17).

### 2.2 Coupled Cluster Calculations

All conventional and explicitly correlated CCSD(T) calculations were performed using the Molpro package.^67−69^ The calculations for open-shell systems correspond to the ROHF-UCCSD(T) formulation^70^ with the (T) term computed as defined in ref (58c). Hartree–Fock (HF) orbitals were used throughout, except in some calculations discussed in Section 3.3 in which Kohn–Sham (KS) orbitals corresponding to the B3LYP exchange–correlation functional were used instead. In these KS-CCSD(T) calculations, the open-shell CC program was used even for singlet states in order to obtain correct (T) terms. In explicitly correlated calculations, we employed the CCSD(T)-F12a and CCSD(T)-F12b formulations by Werner, Knizia and co-workers.^35^ In these calculations, the reference energy includes the CABS singles correction.^35a^ The geminal Slater exponent (γ) was set to 1.0*a*_0_^–1^, except in the calculations performed to accurately recover the TM outer-core correlation correction, in which γ was set to 1.4*a*_0_^–1^ (as recommended for core correlation^47,71^). For the triples term, we considered various scaling schemes, detailed in Results and Discussion. The basis sets and extrapolations are described in Section 2.4.

### 2.3 Multireference Calculations

The NEVPT2^72^ and internally contracted MRCI + Q^73^ calculations were performed using Molpro.^67−69^ The CASPT2 calculations with IPEA-shifted zero-order Hamiltonian^74^ (with the default shift of 0.25 au) and Cholesky decomposition of two-electron integrals^75^ (with the 10^–6^ threshold) were performed using OpenMolcas.^76^ The underlying CASSCF calculations were state-specific, with the exception of the quartet state of , for which the three degenerate components of the ^4^*T*_1*g*_ term were computed together in state-averaged CASSCF, as in ref (66). For NEVPT2, we refer to the partially contracted energies. The size-consistency correction for MRCI + Q is either the Davidson (D) or Davidson–Silver–Siegbahn (DSS) one^77,78^
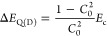
1a
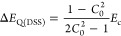
1b(where *E*_c_ is the post-CASSCF correlation energy and *C*_0_ is the coefficient of reference function).

All multiconfigurational calculations are based on the standard choice of active space in mononuclear TM complexes;^79^ i.e., the active orbitals are five 3d orbitals plus one or two (depending on the coordination geometry) metal–ligand bonding orbitals plus three to five correlating orbitals (4d) to describe the double-shell effect, in some cases together with π-backdonation. This choice, leading to a maximum of 12 active orbitals, is similar as in the previous studies of identical or closely related TM complexes.^24,27,65,66^ For , due to strong correlation effects on the Fe=O group, additional π-bonding and correlating orbitals are made active, leading to a somewhat larger active space identical to that used before by Phung, Feldt, and co-workers.^19,20^ Details of the active space choice can be found in Table S1 (Supporting Information). For some complexes, it turned out necessary to reduce the number of double-shell orbitals in lower spin states and/or to apply constraints on the outer-core orbitals (3s or 3s3p) in order to obtain stable active space (see Supporting Information). These procedures are similar to those used in the literature in similar cases.^19,20,65,80^

### 2.4 Basis Sets and Extrapolations

cc basis sets^31,32^ or their combinations are used throughout this article and will be compactly denoted as explained in Table 2 (the notation is similar to that in ref (17)). The cc-pV*X*Z basis set will be denoted as *X*; the corresponding diffuse-augmented set will be denoted as a*X*. The basis set composed of cc-pwCV*X*Z on the TM atom (i.e., containing additional tight functions to describe the TM outer-core correlation effects) and regular cc-pV*X*Z on the ligand atoms will be denoted as c*X*; the corresponding diffuse-augmented set will be denoted as ac*X*. Composite basis sets in which a higher-quality basis (*X*-zeta) is used for the TM atom than for the ligand atoms (*Y*-zeta) will be denoted as *X*/*Y*, e.g., cQ/T or ac5/Q. Reducing the basis set quality to *Y*-zeta on the ligand atoms that are not directly bonded to the TM atom will be indicated with parentheses enclosing *Y*, e.g., cT(D) or cQ/T(D). Note that in all cases, the basis set used for the Cl atom is the (aug)-cc-pV(*X* + *d*)Z^31c^ instead of the regular (aug)-cc-pV*X*Z one, whereas for H atoms, which are mostly spectators for spin-state energetics, the diffuse functions are not added.

**Table 2:** Definitions of Basis Sets Used in This Article

type	examples	atomic basis sets		
		metal^a^	ligand(1)^b^	ligand(2)^c^
*X*	T, Q	cc-pV*X*Z		
a*X*	aT, aQ	aug-cc-pV*X*Z^d^		
c*X*	cT, cQ	cc-pwCV*X*Z	cc-pV*X*Z	
ac*X*	acT, acQ	aug-cc-pwCV*X*Z	aug-cc-pV*X*Z^d^	
c*X*/*Y*	cQ/T, cT/D	cc-pwCV*X*Z	cc-pV*Y*Z	
ac*X*/*Y*	ac5/Q	aug-cc-pwCV*X*Z	aug-cc-pV*Y*Z^d^	
c*X*(*Y*)	cT(D)	cc-pwCV*X*Z	cc-pV*X*Z	cc-pV*Y*Z
c*X*/*Y*(*Z*)	cQ/T(D)	cc-pwCV*X*Z	cc-pV*Y*Z	cc-pV*Z*Z

a TM atom.

b Ligand atoms bonded to TM.

c Ligand atoms not bonded to TM.

d Diffuse functions are not added for H atoms.

In addition to orbital basis sets, the F12 calculations also require auxiliary basis sets (for density fitting and resolution of the identity approximations), which are defined in Table S2 (Supporting Information). The auxiliary bases used in combination with valence-only orbital basis sets (T, Q, aT, and aQ) and with the acT basis set to recover the valence plus TM outer-core correlation effects are analogous as used in ref (47). For the cT, cT(D), and cT/D basis sets used in the economic computational protocol (Section 3.2), the auxiliary bases are analogous as used in our earlier studies.^26,27^

The notation [*X*:*Y*] will be used for a two-point extrapolation to the CBS limit based on the energies calculated with the *X*-zeta and *Y*-zeta quality basis sets. For example, the extrapolation based on the energies calculated using aT and aQ basis sets will be denoted as a[T:Q]. The extrapolation only with respect to the TM’s basis set based on the cT and cQ/T energies will be denoted as c[T:Q]/T, whereas c[T:Q]/T(D) is the analogous one based on the cT(D) and cQ/T(D) energies. The ac[Q:5]/Q extrapolation, which was used to determine the reference CBS limits, is based on the energies from acQ and ac5/Q basis sets.

In conventional (i.e., non-F12) calculations, the correlation energy was extrapolated to the CBS limit using the formula
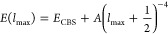
2proposed by Martin^81^ and extensively used by Feller and Peterson with their respective co-workers.^30,39,41,47^ In eq 2, *l*_max_ is the maximum value of the angular momentum quantum number for functions present in the basis set. As pointed out by Bross et al.,^47^ for TM atoms, *l*_max_ = *X* + 1 (where *X* is the cardinal number of the cc basis set), and such a choice was consistently made in this article. The reference energy (HF or CASSCF) was extrapolated using the exponential formula^82^
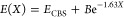
3

For selected results, comparison with alternative extrapolations formulas was made, indicating no major differences (see Supporting Information, Tables S3–S5). In particular, for the ac[Q:5]/Q extrapolation (used to determine the reference CBS limits), different formulas produce indistinguishable results. To extrapolate the CCSD(T)-F12b results, Schwenke-style formula^83^
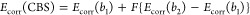
4was applied separately to the CCSD and (T) parts of the correlation energy calculated using basis sets *b*_1_ = aT and *b*_2_ = aQ. The appropriate Schwenke coefficients (*F*) for this pair of basis sets were taken from the work of Hill et al.:^38^*F* = 1.416422 for the CCSD energies or *F* = 1.663388 for the (T) energies. The HF energy (including CABS singles) was taken from the larger basis set.

Note that in all correlated calculations, the inner-core electrons of TM atoms (i.e., 1s2s2p) as well as core electrons of nonmetal atoms (i.e., 1s for the second period, 1s2s2p for Cl) are kept frozen. The outer-core electrons of TM atoms (3s3p) are correlated, except in calculations identified with the prefix FC (frozen core) in which they are frozen.

### 2.5 Relativistic Effects

Most calculations described in this article are nonrelativistic. This choice simplifies the comparison between conventional and explicitly correlated calculations because the F12 methods presently implemented in Molpro can be used only with the nonrelativistic Hamiltonian. Whereas scalar-relativistic effects may contribute a few kcal/mol to the relative spin-state energetics, the relativistic corrections are highly transferable between different theory levels and basis sets;^16,22,25^ thus, they are not expected to affect the basis set convergence to any significant degree. Wherever needed (for comparison with the experiment or results from the literature), the scalar-relativistic corrections were calculated using the second-order Douglas–Kroll (DK) Hamiltonian^84^
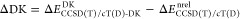
5with conventional CCSD(T). The cT(D)-DK basis set is constructed like the cT(D) one defined above but employing DK-recontractions of the atomic basis sets.^32,85^

## 3 Results and Discussion

### 3.1 Basis Set Convergence of Conventional and Explicitly Correlated CCSD(T)

Our first goal is to carefully study the convergence of spin-state energetics toward the CBS limit in the case of conventional and explicitly correlated CCSD(T) calculations. In this part, we use a similar methodology to that used by Peterson with co-workers in their study of atomization energies for small TM-containing molecules,^47^ in particular, regarding the choice of basis sets. We also perform the analysis separately for the FC CCSD and (T) energies, and the core correlation correction (Δ3s3p), i.e., the three contributions that sum up to the final CCSD(T) energies
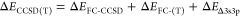
6

Note that the Δ3s3p contribution takes into account only the effects due to the TM outer-core (3s3p) electrons, including both core–core and core–valence correlation terms, and is obtained as the difference of two CCSD(T) energies: the one with valence plus 3s3p electrons correlated, and the one with only valence electrons correlated. For each of the three contributions in eq 6, we separately determine the reference CBS limit and analyze the BSIE as a function of basis set. Detailed inspection of the basis set convergence for each contribution is essential for the main purpose of this work, which is the rational design of a computational protocol: we aim to converge each contribution separately rather than rely on possible cancellations of their BSIEs. Moreover, the breakdown into the FC-CCSD and FC-(T) contributions is advantageous in the context of F12 methods because the explicit correlation is only included for the CCSD energy, whereas the (T) term is computed conventionally.

#### 3.1.1 Reference CCSD(T)/CBS Limits

Nonrelativistic CCSD(T)/CBS limits of spin-state energetics for the studied benchmark set of TM complexes were obtained using the largest affordable extrapolation, ac[Q:5]/Q (see Section 2.4), and are reported in Table 3. All the energy differences are consistently defined as
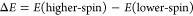
7irrespective of which state is lower in energy, e.g., “^1,5^[Fe(NCH)_6_]^2+^” stands for the *E*(quintet)–*E*(singlet) energy difference.

**Table 3:** Reference CCSD(T)/CBS Limits for Studied Spin-State Energetics^a^,^b^

	HF	FC-CCSD^c^,^d^	FC-(T)^c^	Δ3s3p^e^	CCSD(T)^f^
^1,5^[Fe(NCH)_6_]^2+^	–93.6	–22.6	12.1	3.2	–7.3
^2,6^[Fe(NCH)_6_]^3+^	–107.2	–36.5	10.9	6.5	–19.0
^2,4^[Co(NCH)_6_]^2+^	–53.9	–19.2	5.3	2.5	–11.4
^1,5^[Fe(CNH)_6_]^2+^	–96.9	22.4	22.8	3.6	48.7
^2,6^[Fe(CNH)_6_]^3+^	–98.8	3.5	16.9	7.0	27.5
^3,5^[FeO(NH_3_)5]^2+^	–32.7	–7.1	4.6	2.1	–0.4
^1,5^[Fe(H_2_O)_6_]^2+^	–62.0	–46.0	2.6	–0.4	–43.9
^4,6^[Fe(H_2_O)_6_]^3+^ (ve)	–82.8	–56.6	4.1	4.1	–48.4
^1,3^[FeCp_2_]	–36.3	23.0	10.5	0.8	34.3
^1,3^[FeCp_2_] (ve)	5.8	40.5	7.8	0.3	48.6
^2,6^[MnCp_2_]	–108.6	–22.5	15.5	6.7	–0.4
^1,5^[FeL_2_]	–79.5	–39.3	6.9	1.3	–31.1
^3,5^[FeL_2_] (1)	–41.6	–5.5	6.4	3.3	4.2
^3,5^[FeL_2_] (2)	–42.8	–3.3	7.7	2.9	7.2
^2,6^[FeL_2_Cl]	–103.4	–28.6	15.8	5.3	–7.5
^4,6^[FeL_2_Cl]	–60.5	–14.1	8.1	4.2	–1.8
^1,5^[FeL_2_NH_3_]	–82.2	–24.9	10.7	2.4	–11.9
^3,5^[FeL_2_NH_3_]	–52.9	–9.9	8.7	2.6	1.3

a Values in kcal/mol.

b CBS extrapolation ac[Q:5]/Q using eq 2 for correlation energy and eq 3 for HF energy.

c Only valence electron correlated.

d Including HF energy.

e TM outer-core correlation correction at the CCSD(T) level.

f Sum of the FC-CCSD, FC-(T), and Δ3s3p energies (eq 6).

Note that the FC-CCSD term includes the HF energy. However, for the sake of completeness, HF energies are also reported separately. From the difference of CCSD(T) and HF energies, one can easily calculate the differential correlation energies, which are very significant (from 18 to 146 kcal/mol, with the median 56 kcal/mol) and uniformly positive. The positive sign indicates (under the sign convention of eq 7) that the electron correlation stabilizes a lower-spin state more considerably than a higher-spin state, which agrees with similar observations in the literature^16,22^ and is intuitive because there are more paired electrons in the lower-spin state. In other words, the recovery of electron correlation reduces the strong HF bias toward higher-spin states by making the Δ*E* values greater (i.e., less negative or more positive) than at the HF level. The differential correlation effects observed here should be regarded as very large in magnitude, especially considering that during these spin-state transitions, the number of unpaired electrons changes by only two or four (depending on the Δ*S* value), whereas the chemical bonds are preserved. We further note that about 80% of the differential correlation energy is recovered at the FC-CCSD level and the FC-(T) term comprises about 12–18% of the correlation energy. The triples thus contribute significantly to the spin-state energetics (they also contribute through the Δ3s3p term). The Δ3s3p term, typically several times smaller than the FC-(T) term, is sized up to 7 kcal/mol (median: 3 kcal/mol) and is uniformly positive with one exception.

The chosen reference extrapolation, ac[Q:5]/Q, is the largest affordable one and is identical with the reference extrapolation used in the study of Phung et al.^19^ (at the CASPT2 level). For the presently studied systems, the chosen ac[Q:5]/Q extrapolation gives a difference in relative CCSD(T) energies up to about 1 kcal/mol in comparison with the smaller, all-atom ac[T:Q] extrapolation (Table S6, Supporting Information). The use of fixed, quadruple-ζ basis set on the ligands in the ac[Q:5]/Q extrapolation is an approximation, which would be clearly insufficient for general thermochemical applications but is justified here for spin-state energetics, being strongly dominated by the TM atom contribution. Another, indirect argument supporting the accuracy of the present reference CBS limits is their good agreement with the explicitly correlated results (at the CCSD-F12b level), as discussed in Section 3.1.2.

#### 3.1.2 FC-CCSD Energy

**Figure 2:**
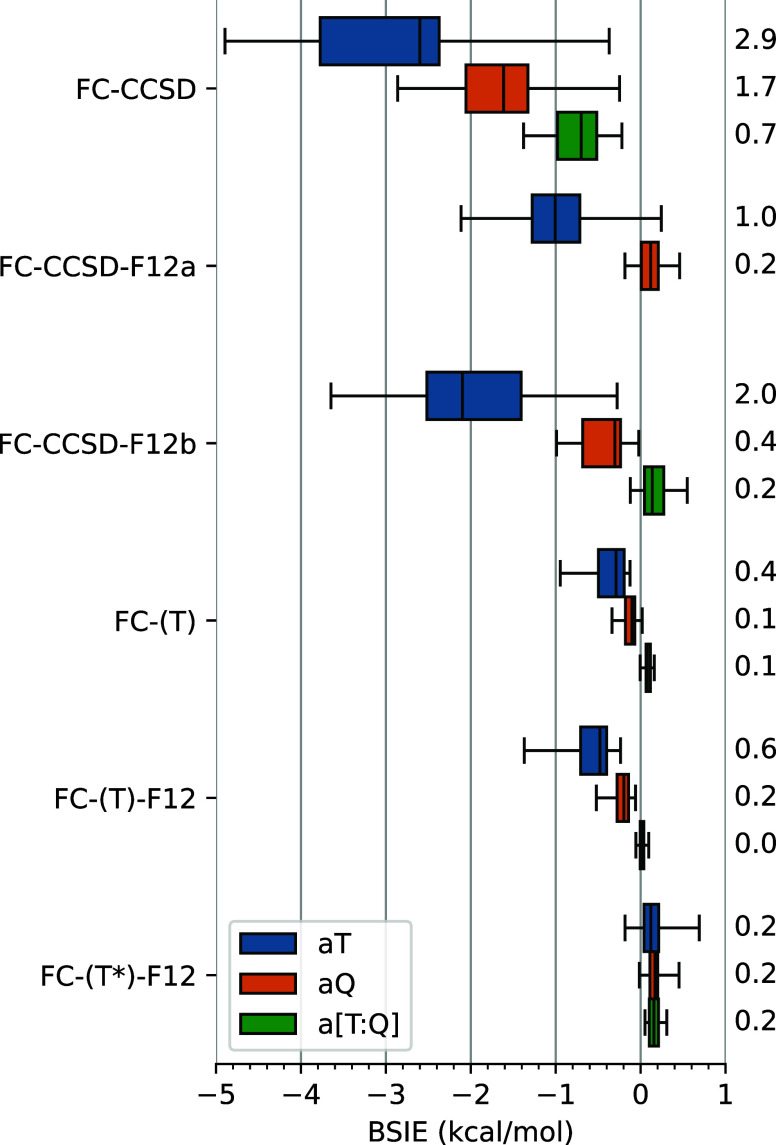
Box-plot of basis set incompleteness errors (BSIEs) of the FC-CCSD and FC-(T) terms in conventional and explicitly correlated calculations relative to the CBS limits from Table 3. Each box represents 50% of the population (with the median marked in the middle), and the whiskers extend from the minimum to the maximum. Annotated values are MADs (mean absolute deviations). See Supporting Information for numeric data of individual complexes (Tables S7 and S8) and the timings of representative calculations (Figure S1).

Using the reference CBS limits of Table 3, we now analyze the performance of conventional and explicitly correlated (F12a/b) calculations in recovering the FC-CCSD contributions to spin-state energetics (Figure 2, top). Similarly to the approach used in ref (47), the FC energies were calculated using valence-only, diffuse-augmented cc basis sets of the triple-ζ and quadruple-ζ quality, briefly denoted as aT and aQ; the CBS extrapolation based on these two basis sets is denoted a[T:Q] (see Section 2.4 for details of the basis sets and extrapolations).

As can be easily seen, the conventional FC-CCSD energies converge slowly to the CBS limit, with the observed MAD (mean absolute deviation) of 2.9 kcal/mol for the aT basis set and still 1.7 kcal/mol for the aQ one; the maximum errors are by ∼70% larger. Even after the a[T:Q] extrapolation, the BSIEs are still relatively large (MAD 0.8, maximum 1.4 kcal/mol). This is not changed much by using alternative extrapolation formulas, including the Truhlar-type^33,86^ or Schwenke-type^83^ extrapolations, which contain parameters that have been optimized to reproduce accurately estimated CBS limits for small molecules or atoms (Table S5, Supporting Information). The slow basis set convergence of conventional FC-CCSD spin-state energetics resembles that observed for atomization energies of small TM molecules in ref (47).

The FC-CCSD-F12a/b energies approach the CBS limits more rapidly. When extrapolated to the CBS limit using Schwenke-style eq 4, the FC-CCSD-F12b energies have negligible BSIEs (MAD 0.2, max 0.5 kcal/mol). The analogous extrapolation is not performed for the FC-CCSD-F12a energies due to the lack of corresponding Schwenke coefficients. However, with a given basis set, the FC-CCSD-F12a results exhibit smaller BSIEs than the corresponding F12b results. Actually, the F12a results using the aQ basis set are as good as the F12b results after the extrapolation. This superior performance of the F12a formulation, especially when using small basis sets, was also noted for atomization energies and ascribed to favorable error cancellation.^41^ We will employ this feature later in design of an efficient computational protocol (Section 3.2).

We further note that in the case of conventional FC-CCSD calculations, all the BSIEs are systematically negative, i.e., the spin-state energetics converge to the CBS limit from below. Taking into account our sign convention for energy differences (eq 7) and noticing that correlation effects are greater in the lower-spin state, this behavior is indicative of underestimating the correlation effects in a finite basis set with respect to the CBS limit. Interestingly, such a regular behavior is not always observed in F12 calculations; in particular, neither for the F12a/aQ energies nor for the a[T:Q]-extrapolated F12b energies. Slightly positive BSIEs observed in these F12 calculations are indicative of a minor overestimation of the correlation effects, but this effect is small and will have limited practical consequences. In fact, these slightly positive BSIEs are so small that they are likely comparable to unavoidable uncertainties in our reference CBS limits for the FC-CCSD energy. (Obtaining definitely more accurate CBS limits is problematic unless one could perform extremely expensive calculations with a sextuple-ζ basis set, which is anyway not available for TM atoms.) Therefore, at this stage, it is most appropriate to conclude that the best available FC-CCSD-F12 energies, namely, the F12a/aQ or the F12b/a[T:Q] ones, are of comparable quality as our reference FC-CCSD/CBS limits, and they agree with each other to within 0.5 kcal/mol or better. The good mutual agreement between the best available F12 and conventional energies can be treated as an argument supporting the accuracy of both of them.

#### 3.1.3 FC-(T) Energy

As further shown in Figure 2, bottom, the FC-(T) correction to spin-state energetics introduces a BSIE which is usually several times smaller than the one rooted in the FC-CCSD energy. In particular, when using conventional a[T:Q] extrapolation, the FC-(T) term is excellently converged to the CBS limit (MAD 0.1, max 0.2 kcal/mol). Even with the smallest basis set considered here (aT), the BSIE in the conventionally calculated FC-(T) term is within 1 kcal/mol. Considering now the FC-(T) energies from the F12 calculations (which are identical in the F12a and F12b variants), one should remember that the triples correlation energy does not directly benefit from the explicit correlation treatment. The only reason why slightly different (T) energies are obtained from the conventional and F12 calculations is the difference in the underlying singles and doubles amplitudes. The results in Figure 2 show that FC-(T)-F12 energies do not perform systematically better than conventional ones, especially with the aT basis set, where we observe the BSIEs reaching 1.4 kcal/mol. This accounts for a substantial contribution to the overall BSIE, when compared with the BSIE of the FC-CCSD-F12a/b term using the same basis set, due to the aforementioned lack of the F12-based treatment for the triples. With the aim of reducing the BSIE in the (T) term, Werner with co-workers proposed^35b,87^ to scale it as
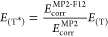
8i.e., the scaling coefficient is the ratio of correlation energies from explicitly correlated and conventional MP2 calculations. Eq 8, also referred to in the literature^42^ as Marchetti–Werner approximation, is based on the assumption that the MP2 and (T) correlation energies converge to the CBS limit with a similar regularity and has been employed mainly in the context of atomization energies and noncovalent interactions. Our data for spin-state energetics show that the (T*) scaling procedure indeed improves the accuracy for the smaller basis set (aT), but there is no evident improvement for the larger basis set (aQ). Finally, if the a[T:Q] extrapolation is concerned, the unscaled FC-(T)-F12b energies are excellently converged to the CBS limit, and hence, their scaling with eq 8 is not beneficial at all (in fact, it slightly increases the BSIEs, although the deterioration is very small). The present finding is somewhat different from the observations in ref (47) for atomization energies of small TM-containing molecules, where it was found that the (T*) correction outperforms the unscaled (T) one also in the quadruple-ζ basis set.

#### 3.1.4 Core Correlation Term, Δ3s3p

**Figure 3:**
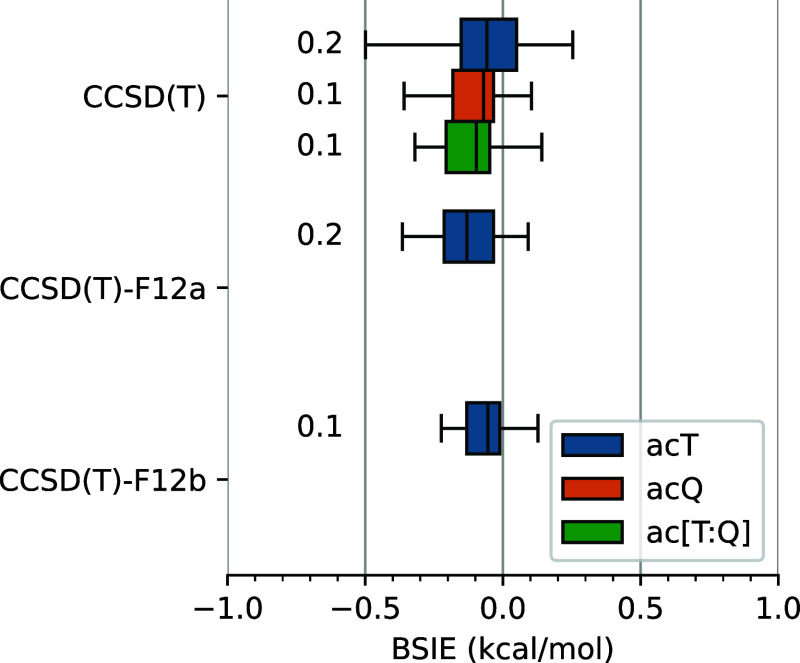
Box-plot of basis set incompleteness errors (BSIEs) of the Δ3s3p term in conventional and explicitly correlated CCSD(T) calculations relative to the CBS limits from Table 3. The graphical convention is identical to that used in Figure 2 (but mind the different scale); the annotated values are MADs. See Supporting Information for numerical data of individual complexes (Table S9) and the timings of representative calculations (Figure S1).

The BSIEs in the Δ3s3p term at the CCSD(T) level are shown in Figure 3. The results of conventional and explicitly correlated calculations are considered in the acT basis set; for the conventional calculations, additionally, acQ and extrapolated ac[T:Q] results are shown. (The basis sets acT and acQ contain additional tight functions on the TM atoms to describe the outer-core correlation effects; see Section 2.4 for details.) It is clear that the results are converged to within 0.5 kcal/mol already in conventional calculations with the acT basis set. By using the ac[T:Q] extrapolation or employing the F12b methodology with the acT basis set, the BSIE can be reduced further to reach an excellent accuracy (MAD 0.1, max 0.2 kcal/mol).

Thus, for presently studied spin-state energetics, the Δ3s3p correction is the easiest of the three terms in eq 6 to converge with the basis set. Comparing with the case of atomization energies for small TM-containing molecules,^47^ we notice that the Δ3s3p corrections for spin-state energetics appear to converge somewhat faster with basis set than in the case of atomization energies (in terms of the maximum and average deviations observed), even if the Δ3s3p corrections are comparable in magnitude for the two cases.

### 3.2 Efficient Computational Protocol Based on CCSD(T)-F12a

#### 3.2.1 Motivation

The analysis in the previous section confirms that the CCSD(T)/CBS limits of spin-state energetics can be accurately recovered using an explicitly correlated methodology, for example, by combining the a[T:Q]-extrapolated FC-CCSD(T)-F12b energies with core correlation corrections calculated at the CCSD(T)-F12b/acT level, i.e.,
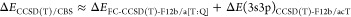
9

This is similar to the strategy adopted by Peterson and co-workers in their study of atomization energies for small TM molecules.^47^ The use of F12b ansatz is favored over F12a due to its systematic basis set convergence with large basis sets. However, the calculations based on eq 9 become prohibitively expensive for larger TM complexes due to need of performing for each spin state very demanding calculations with the aQ (diffuse-augmented quadruple-ζ) basis set and three more calculations with triple-ζ basis sets. Therefore, in this section, we design an alternative explicitly correlated protocol tailored to approximate the CCSD(T)/CBS limits of spin-state energetics at a significantly reduced computational cost.

The design of such a protocol is based on the following premises. First, recognizing that in larger TM complexes it would be impractical to use a larger than triple-ζ basis set, we choose the explicitly correlated approach and focus on the CCSD-F12a ansatz due to its superior performance with small basis sets (see the previous section). Second, as the presence of diffuse functions considerably increases the computational cost, we consider the possibility of not augmenting the basis set. Third, having learned above that the outer-core correlation corrections to spin-state energetics quickly converge with basis set size, we consider the possibility of treating valence-only and TM outer-core correlation effects together in single calculations to reduce the number of calculations that have to be performed. Finally, due to the relatively localized nature of the considered spin-state transitions, we consider the possibility of reducing the basis set on less important ligand atoms.

#### 3.2.2 Construction

**Figure 4:**
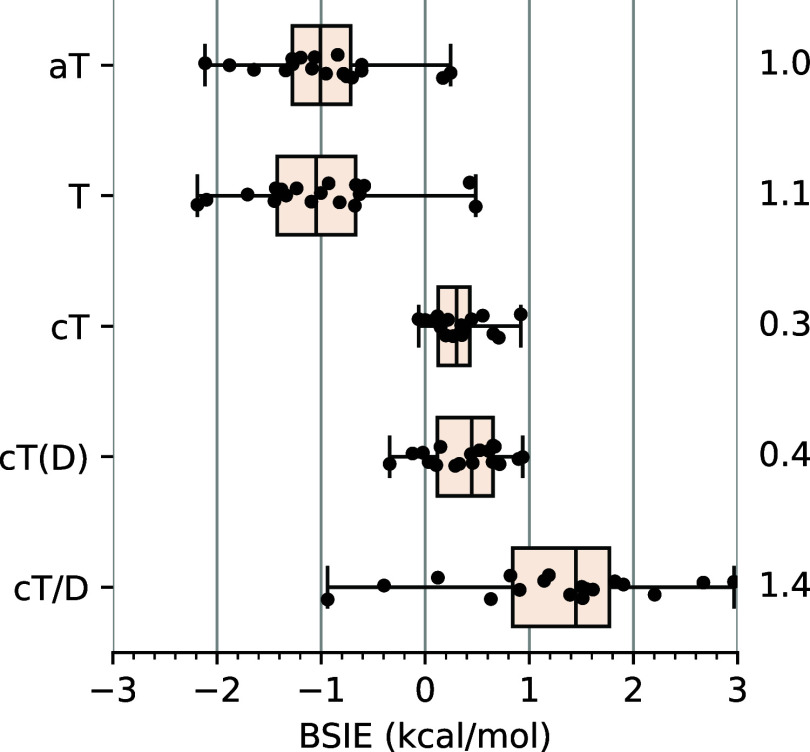
Box-plot of basis set incompleteness errors (BSIEs) in FC-CCSD-F12a calculations with several basis sets of triple-ζ (or lower) quality relative to the CBS limits of Table 3. Individual data are represented with points. Annotated values are MADs. See Supporting Information for numeric data (Table S10) and the timings of representative calculations (Figure S2).

We start the construction of a computationally efficient CCSD(T)/CBS approximation by analyzing the FC-CCSD energy, which is typically the largest contributor to the BSIE (see the previous section). Figure 4 shows the statistical distribution of the BSIEs for the FC-CCSD-F12a energies computed with several basis sets of triple-ζ quality or smaller (for the notation of basis sets, see Section 2.4.). The first two basis sets are valence-only triple-ζ ones with (aT) or without (T) the diffuse functions. The third basis set (cT) contains additional functions in the TM core region, which are needed to properly describe the 3s3p correlation. (But note that the 3s3p electrons are not correlated at this stage. We consider such a basis set in the FC-CCSD calculations with the idea of later being able to describe all correlation effects with one basis set.) Finally, the last two basis sets are modifications of the cT basis in which it is reduced to double-ζ quality either on all ligand atoms (cT/D) or on the atoms not directly bonded to the TM atom (cT(D)).

By comparing the results obtained with the aT and T valence-only basis sets, it becomes clear that the augmentation hardly improves the accuracy of computed spin-state energetics. This result is somewhat in contrast to previous observations that “diffuse basis functions in the augmented correlation consistent basis sets are important both for obtaining accurate HF values as well as for the F12 correlation treatment. Non-augmented basis sets should therefore not be used in F12 calculations”,^35b^ which were made upon studies of small molecules and specifically in the context of atomization energies, ionization potentials, and electron affinities. Further comparison in Table S11, Supporting Information, demonstrates that for spin-state energetics, the effect of adding diffuse functions is even smaller in the F12 than in the conventional FC-CCSD calculations. The presently observed unimportance of diffuse functions in the F12 calculations of spin-state energetics can be rationalized by noting that the change of spin state is localized on the TM center with the nearest neighborhood, i.e., in the region of strong overlap between the metal’s and ligand’s basis functions, whereas the long-range behavior of orbitals is less important.

By contrast, switching from the T to the cT basis set turns out to be very important, and actually, it brings a qualitative improvement in the accuracy of the FC-CCSD-F12a energies: the maximum and mean absolute BSIEs drop to only 0.9 and 0.3 kcal/mol, respectively, i.e., they are reduced by the factor of 2–3 compared with those for the valence-only basis sets (T and aT). It was further checked that the difference of 1–3 kcal/mol between the FC-CCSD energies calculated with the T and cT basis sets is observed not only in explicitly correlated calculation but also in conventional ones and also for bare metal ions surrounded by point charges (Table S12, Supporting Information). It appears, therefore, that the additional radial flexibility in the TM core region introduced in the cT basis set is beneficial for the recovery of the valence-only correlation effects that are relevant to relative spin-state energetics. While this result may seem at first sight somewhat counterintuitive, it is reminiscent of the compactness of the TM valence shell, i.e., the similarity of spatial extents for the 3d and 3s3p atomic orbitals. In calculations using quadruple-ζ or larger basis sets, or extrapolated to the CBS limit, the benefits from this additional radial flexibility of the core-augmented basis set in the valence region will be minor or negligible.^88^ Here, however, when the basis set is by construction restricted to the triple-ζ quality, one cannot ignore the importance of observation that the FC-CCSD-F12a spin-state energetics are systematically closer to their CBS limits if the core-augmented basis set is assigned to the TM atom. Slightly positive BSIE values obtained with the cT basis set are reminiscent of the tendency of the F12a ansatz to overestimate the correlation energy (see above). While this feature may be disgusting from the purist’s point of view, it is actually helpful to obtain chemically accurate energy differences with the triple-ζ basis set. The relatively small values of the mean and median BSIE value as well as the narrow distribution of errors for the FC-CCSD/cT calculations, which are clearly seen in Figure 4, confirm that this approximation, even if rooted in some error cancellation, is quite systematic in the case of TM spin-state energetics and hence worthy of being employed in the construction of an efficient computational protocol.

Finally, considering the possibility of basis set reduction on part of the ligand atoms, we note that the calculations with the cT(D) basis set—i.e., reduced on the ligand atoms except those bonded to the TM atom—have obviously slightly larger BSIEs than full cT calculations, but the resulting errors are still relatively small (MAD 0.5, max 1 kcal/mol), keeping in mind the chemical accuracy and a significantly lower cost of the calculations with such a reduced basis set (especially if the triples are included later; see Figure S2, Supporting Information). Further reduction to the cT/D basis set leads to significantly larger BSIEs (up to 3 kcal/mol) and is therefore not recommended. These results are in accord with the chemical intuition: the change of spin state affects electronic structure, mostly the metal and its nearest neighbors, making the quality of the basis set on these parts of the molecule far more important than on the outer-lying ligand atoms. On the other hand, the usage of triple-ζ one the nearest ligand atoms is critical because the change of spin state propagates through the metal–ligand bonds.^52^

**Figure 5:**
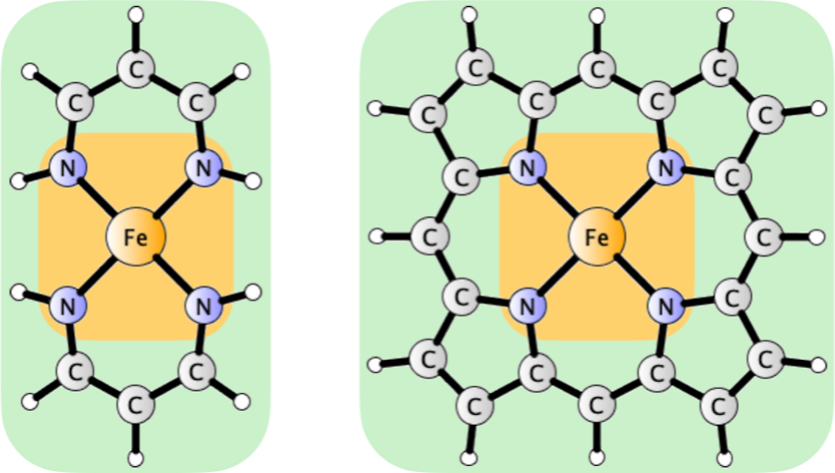
Graphical representation of the composite basis set cT(D) for the example of one of the small models studied here (Fe^II^L_2_, left) and Fe^II^ porphyrin complex (FeP, right). The colored areas represent the assignment of atomic bases: triple-ζ for Fe and coordinated N atoms (orange) and double-ζ for the other atoms (green). Note that the number of atoms in the triple-ζ region does not increase with expansion of the ligand.

Overall, the usage of the cT(D) basis set appears to be a good trade-off between the accuracy and computational cost. The savings from reducing the basis set on the outer-lying ligand atoms will be particularly significant for complexes with expanded organic ligands, such as real SCO complexes or metalloporphyrins (Figure 5), for which CC calculations with the full triple-ζ basis set would be very expensive or simply intractable. Obviously, the accuracy of this approximation relies on the locality of changes in the electronic structure caused by the change of the metal’s spin state. The accuracy is expected to degrade in the case of complexes with noninnocent ligands, but none of the complexes studied here are like that.

Having decided on the usage of the cT(D) basis set, we subsequently analyze the BSIEs in the FC-(T) energy obtained from F12a calculations. As discussed above, the (T) term does not benefit from the explicit correlation treatment, and therefore, the scaled (T*) term (Marchetti–Werner approximation, eq 8) was proposed to approximately correct this deficiency. Here, we consider a more general form of the triples correction
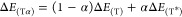
10which is a linear interpolation between the (T) and (T*) correlation energies controlled with the mixing parameter α. The usage of such (Tα) term does not require any additional calculations beyond the standard CCSD(T*)-F12a, only different postprocessing of the results (see the sample input file provided in Supporting Information, Section S7).

**Figure 6:**
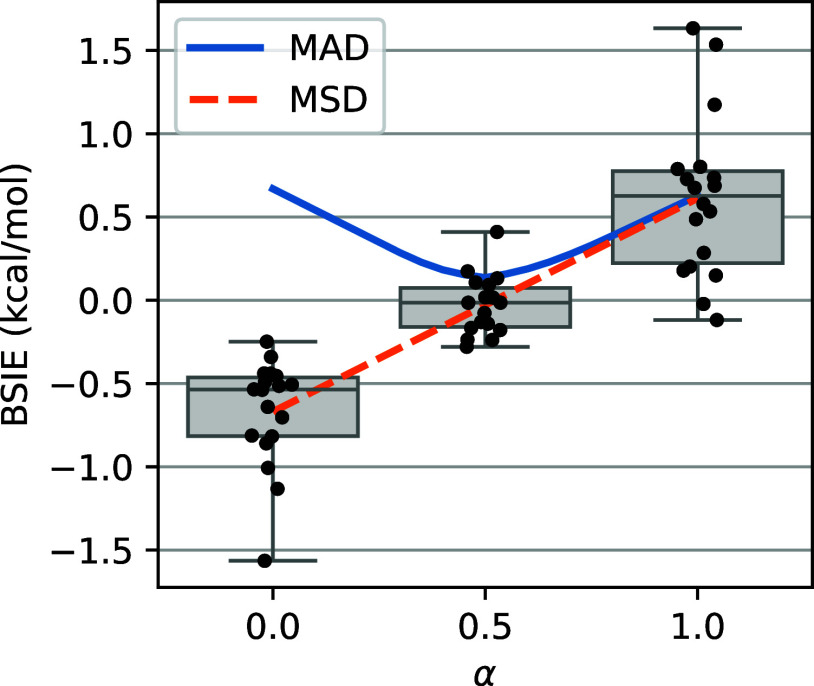
Basis set incompleteness error (BSIE) of the FC-(Tα) term of eq 10 for the spin-state energetics computed at the CCSD(T)-F12a/cT(D) level. The mean absolute deviation (MAD) and mean signed deviation (MSD) from the reference values are plotted as functions of α, and statistical box-plots are shown for the three representative values: α = 0 corresponding to the unscaled (T) correction, α = 1 corresponding to the Marchetti–Werner (T*) correction, and  corresponding to the (T#) correction proposed in this work. For numerical data of individual complexes, see Table S13, Supporting Information.

Figure 6 shows the BSIEs in the FC-(T) contribution to the studied spin-state energetics based on the linear interpolation model (Tα) of eq 10 as a function of the α parameter between 0 and 1. Considering the distribution of errors, it is clear that the optimum choice of α for the presently studied spin-state energetics is neither 0 nor 1, but rather something in between. With α = 0 (i.e., bare unscaled triples), the observed BSIEs are systematically negative, indicating the underestimation of the correlation energy, whereas with α = 1 (i.e., the Marchetti–Werner scaling), the BSIEs tend to be positive. Moreover, relatively large outliers are observed for α = 0 and 1, showing that for the (T) or (T*) formulations, the FC-(T) term is in some cases a more important contributor of the BSIE than the FC-CCSD energy. The choice of value  leads to a nearly zero mean signed deviation (MSD) and is very close to the minimum of the MAD. Moreover, this choice narrows the error distributions and considerably reduces the outliers. Therefore, the triples correction of eq 10 with  appears to be the best choice for spin-state energetics calculated using the cT(D) basis set, and this new formulation will be denoted as (T#).^89^

The last term to be considered is the Δ3s3p correction. As might be expected based on the previous results, the Δ3s3p term is almost converged to the CBS limit at the CCSD(T)-F12a/cT(D) level. Moreover, the Δ3s3p term is almost insensitive to the applied variant of the triples correction [unscaled (T), (T*), or (T#)] and the geminal exponent value (γ = 1.0 vs 1.4*a*_0_^–1^); see Table S14, Supporting Information. For simplicity, we choose the (T#) variant with γ = 1.0. With this choice, one can recover the valence plus TM outer-core correlation effects together from single CCSD(T#)-F12a calculations in which the 3s3p and valence electrons are correlated jointly.

Based on these considerations, we propose the following computationally efficient protocol to approximate the CCSD(T)/CBS limits of TM spin-state energetics
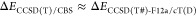
11where (T#) is defined by eq 10 with . Its performance is discussed below.

#### 3.2.3 Performance and Efficiency

**Figure 7:**
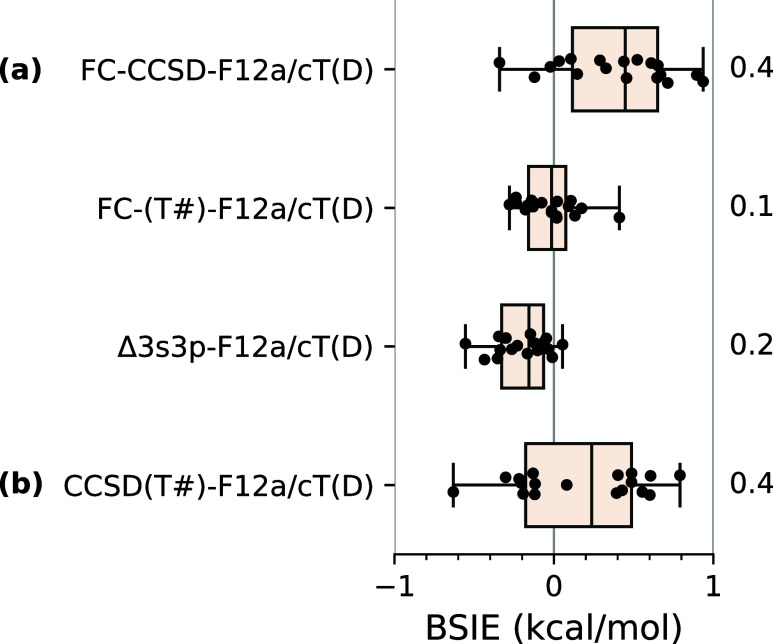
Basis set incompleteness errors (BSIEs) for the spin-state energetics calculated at the CCSD(T#)-F12a/cT(D) level with respect to the reference CBS limits of Table 3: (a) for the FC-CCSD, FC-(T#), and Δ3s3p terms separately and (b) for the final CCSD(T) energy estimates, i.e., sums of the above three terms. Values annotated to the right are MADs.

Figure 7 reports the distribution of BSIEs for the approximation defined in eq 11 with a breakdown into the separate contributions discussed above: FC-CCSD-F12a, FC-(T#)-F12, and Δ3s3p-F12a terms. In can be seen that all three contributing terms as well as the final estimates at the CCSD(T) level are converged to the respective CBS limits with the accuracy of 1 kcal/mol or better. The final energy estimates have the MAD of only 0.4 kcal/mol.

If the interest is only in estimating the CCSD(T)/CBS limits, there is obviously no reason to separately compute the three contributing terms [FC-CCSD, FC-(T), and Δ3s3p]. In fact, an important advantage of the computational protocol in eq 11 is the simplicity: only one explicitly correlated calculation has to be performed for each state. However, with the focus on the protocol’s transferability and universality, it is important to note that the approximation defined in eq 11 performs so well because each term is well converged to its own CBS limit (cf. Figure 7a). In addition, there is some tendency to favorable cancellation of slightly positive errors in the FC-CCSD energy with slightly negative errors in the Δ3s3p correction. In principle, one might consider to further improve the accuracy by optimizing the α parameter in eq 10 to minimize the BSIEs for the CCSD(T#)-F12a/cT(D) energy differences, not just for only the FC-(T) term. However, we refrain to do so in order to avoid excessive empiricism. Moreover, if such an approach is attempted, the minimum on the resulting MAD curve as a function of the α parameter is very broad (see Figure S3, Supporting Information), and hence, there is actually no gain from changing α.

We note in passing that F12 calculations with the cT(D)-type basis sets, similar to the protocol defined in eq 11, were already performed in our previous studies of spin-state energetics.^8,27,66^ The choice of molecular-orbital and auxiliary basis sets was inspired by the earlier study of Harvey and co-workers,^49^ who pioneered the application of CCSD(T)-F12 methods to open-shell TM complexes. However, it is first time here that the protocol has been formalized and undergone systematic assessment. Moreover, an important new development is the modified triples correction, (T#), which has been demonstrated to perform considerably better than either the (T) or (T*) term used before (see also Table S15, Supporting Information).

**Figure 8:**
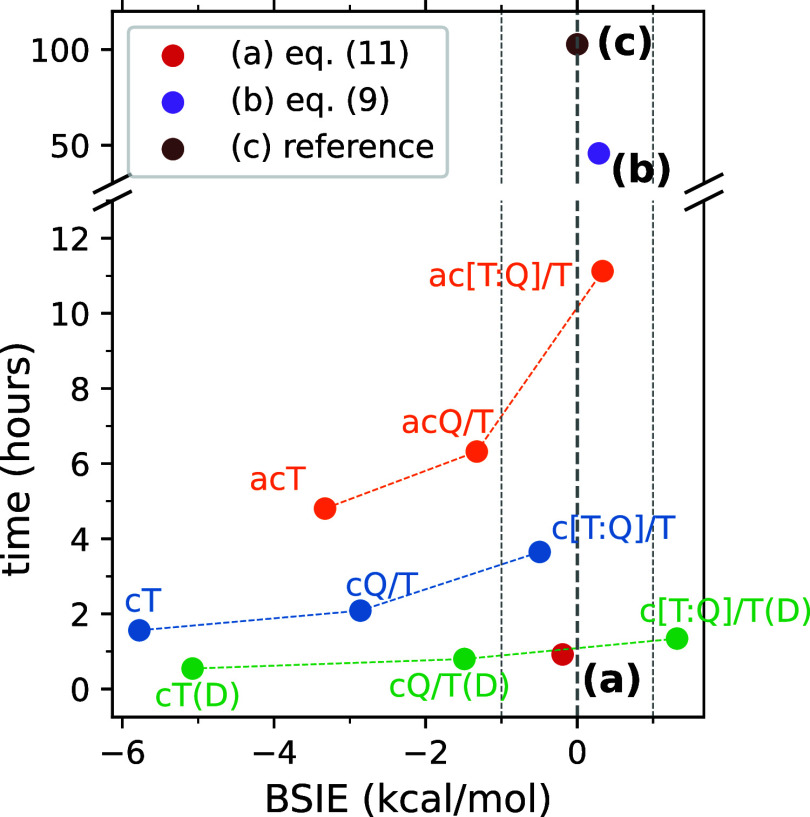
Relation between the computation time and basis set incompleteness error (BSIE) resulting from calculations of the quintet–singlet energy difference for  at the CCSD(T) level: (a) the CCSD(T#)-F12a/cT(D) protocol of eq 11 (b) the CCSD(T)-F12b/a[T:Q] + Δ3s3p-F12b/acT protocol of eq 9 and (c) the reference CBS extrapolation ac[Q:5]/Q, and some other conventional CCSD(T) calculations and extrapolations (see Section 2.4 for the notation of basis sets and extrapolations). The reported times are obtained by summing the wall clock times needed to compute the energies of the two spin states. All times were measured using Molpro 2019.2 running 12 MPI processes, each allocating up to 24 GB of RAM, on an Intel Xeon 6146 system equipped with triple RAID-0 of Intel P4600 solid-state drives.

In order to illustrate efficiency of the proposed computational protocol, Figure 8 shows the relation between the computation time and the BSIE obtained for the quintet–singlet splitting of . It is clear that the computational protocol of eq 11 provides an excellent balance between the accuracy (small BSIE) and efficiency (small computation time). The computation time is ca. 40 times smaller than that for a more systematic F12 approach of eq 9 and ca. 100 times smaller than that for the reference CBS extrapolation. These differences will be even more pronounced for larger complexes. Conventional CBS extrapolations can give comparably small BSIEs only at the expense of larger computation times. The extrapolations with the triple-ζ basis set on ligand atoms, such as c[T:Q]/T or ac[T:Q]/T, achieve comparable accuracy, but they are several to 10 times more expensive. A more economic extrapolation procedure c[T:Q]/T(D), in which the ligand basis set is reduced to the double-ζ on the C and H atoms not bonded to Fe, is only slightly more expensive, but it gives a larger BSIE. More systematic comparison with economic extrapolation protocols will be given in Section 3.4.

### 3.3 Transferability of Basis Set Incompleteness Error

So far, we were dealing with BSIEs for spin-state energetics computed with the CCSD(T) method and proposed the economic computational protocol to make these BSIEs small enough. We are now interested in comparing the BSIEs that are obtained from different methods when using a common basis set. Let us denote an energy difference calculated using method *m* in the finite basis set *b* as Δ*E*_*m*/*b*_ and the analogous value in the CBS limit of method *m* as Δ*E*_*m*/CBS_. The BSIE of method *m* when using basis set *b* is then defined as
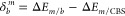
12

In this analysis, we stick to the basis set cT(D), i.e., the one that was used for the economic F12a protocol in Section 3.2, and calculate spin-state energetics with a number of methods in addition to CCSD(T) discussed above: KS-CCSD(T) (using B3LYP orbitals), MP2, CASPT2, CASPT2/CC,^19^ NEVPT2, and MRCI+Q with two variants of the size-consistency correction. (One of complexes, , was excluded from the MRCI+Q and NEVPT2 calculations due to prohibitively large size of its active space.) For all methods, the reference CBS limits were obtained from the ac[Q:5]/Q extrapolation, i.e., the same one as used above for the CCSD(T). The results in the CBS limit and in the cT(D) basis set are reported in Tables S16 and S17 (Supporting Information). The two sets of results differ noticeably due to large BSIEs, which are systematically negative, sized up to 7 kcal/mol, but more typically 3–4 kcal/mol. It is also evident from these results that spin-state energetics predicted by different WFT methods diverge much beyond the chemical accuracy.

**Figure 9:**
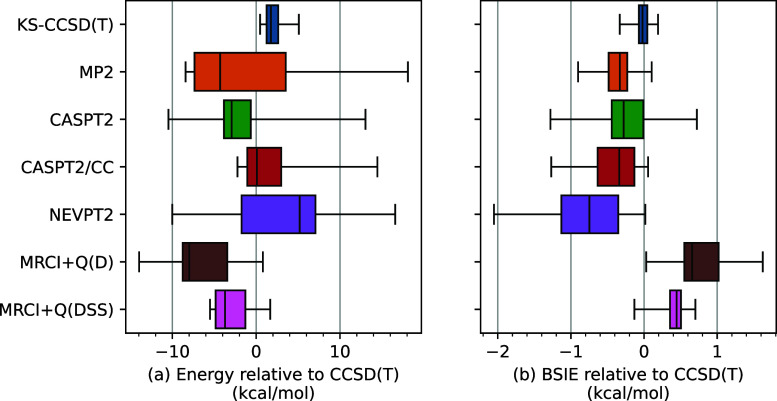
Statistical distribution of (a) differential energies between various methods and CCSD(T) in the CBS limit and (b) the corresponding differential BSIEs obtained using the cT(D) basis set for the studied benchmark set of spin-state energetics. Mind different energy scales for (a,b). For numerical data of (b), see Table S18, Supporting Information.

In order to analyze the discrepancies between different methods, Figure 9a shows the distribution of energy differences relative to the CCSD(T) ones, i.e., the quantities Δ*E*_method/CBS_ – Δ*E*_CCSD(T)/CBS_. The discrepancies shown in panel (a) are very significant; for example, in the case of MP2, they fluctuate from −8 and 18 kcal/mol. This was to be expected as MP2 energies are very bad approximations to the CCSD(T) ones for TM complexes.^61^ Comparably large deviations from the CCSD(T) energies are observed for CASPT2 and NEVPT2, whereas somewhat smaller, yet still chemically significant, deviations are observed in the case of MRCI+Q and KS-CCSD(T). By contrast with this, panel (b) of Figure 9 shows the distribution of BSIEs of different methods relative to the CCSD(T) ones, i.e., the quantities δ_cT(D)_^method^ – δ_cT(D)_^CCSD(T)^. It follows that, strikingly, all methods give BSIEs rather similar to those of the CCSD(T) method, often identical to within ±1 kcal/mol. The best agreement with the CCSD(T)’s BSIEs is observed for KS-CCSD(T), MP2, and MRCI+Q(DSS). Even in the worst cases of NEVPT2 and MRCI+Q(D), the discrepancies between the BSIEs of a given method and those of the CCSD(T) tend to be several times smaller than the discrepancies between the raw energies (panel a) and rarely exceed 1 kcal/mol (the largest deviation is −2.1 kcal/mol for NEVPT2). Although this analysis arbitrarily singled out the CCSD(T) method, a good to excellent correspondence can be observed between the BSIEs of all WFT methods considered here, which is shown by the correlation matrix of the BSIEs (Figure S5, Supporting Information).

A condition that the BSIEs of different methods *m* and *n* are approximately equal, i.e.,
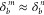
13shall be called here the BSIE transferability. As just shown in Figure 9b, the BSIEs for spin-state energetics computed using the cT(D) basis set are very well transferable between CCSD(T) and other methods considered: to within 0.3 kcal/mol for KS-CCSD(T); to within 1 kcal/mol for MP2 and MRCI+Q(DSS); or to within 2 kcal/mol for the remaining methods. It must be reiterated that the BSIE transferability observed here originates neither in similarity of the results obtained from different methods (because they are not similar) nor in smallness of the BSIEs (because they are not small). It rather comes from the fact that BSIE is approximately independent of the method’s IE, and hence, even methods giving different results may have similar BSIEs.

The concept of BSIE transferability is closely related to the approximations made in composite thermochemical models^88,90−93^ [e.g., Gaussian-n, Weizmann-n, HEAT, Feller–Peterson–Dixon, and correlation consistent composite approach (ccCA)] and closely related focal point approaches,^94−98^ namely, that energy contributions from higher-order correlation effects can be evaluated using a smaller basis set than contributions from lower-order effects.^99^ In other words, the CBS limit for a higher-level method *m* can be approximated using the CBS limit for a lower-level method *n*
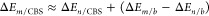
14i.e., by assuming that the difference between the methods’s CBS limits can be additively corrected using the difference between their results in the finite basis set *b*. Reordering of eq 14 and recognizing the definition of the BSIE from eq 12 immediately leads back to eq 13, showing that the additive approximation in eq 14 is equivalent to the BSIE transferability condition. The validity of this approximation is often not possible to directly verify in large systems, because high-level calculations with large basis sets are often too expensive to perform. For the presently studied set of spin-state energetics, we have just shown above that eq 13 holds to a good approximation.

The above observations support the following focal-point approximation to the CCSD(T)/CBS spin-state energetics based on the computationally cheap MP2 method to estimate the BSIE
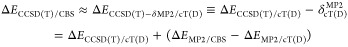
15

This is similar to the approximation proposed by Dunning and Peterson^99^ as well as those made in the ccCA method of Wilson and co-workers^46,93^ and introduced for intermolecular interactions.^97,98,100^ With regard to TM complexes, there have been presumptions in the literature that such an approximation cannot correctly recover the CCSD(T)/CBS limits due to the “disastrous behavior of MP2 for TM complexes”.^61^ Actually, however, our data in Figure 9b demonstrate that this approximation is reliable to within 0.9 kcal/mol (MAD 0.4 kcal/mol) for the studied benchmark set of spin-state energetics.

Conversely, if the CCSD(T)/CBS limits were approximated using the efficient CCSD(T#)-F12a protocol described above, they can be used to approximate the CBS limit of another method *m* from the BSIE transferability condition, i.e.
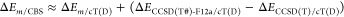
16where the term in bracket serves to correct the BSIE from the small basis set result. This is a very convenient approximation if many methods are to be tested, and indeed, we have already employed a similar approach in our previous benchmark studies^26,27^ [where it was introduced on an intuitive basis and with a notable difference that the CCSD(T*)-F12a energies were used rather than the presently introduced, more accurate CCSD(T#)-F12a ones]. It can be verified for the present set of spin-state energetics that the additive approximation in eq 16 is accurate to within 1 kcal/mol for KS-CCSD(T), CASPT2, and CASPT2/CC (MAD 0.4 kcal/mol); to within 1.5 kcal/mol for MRCI+Q (MAD 0.6–0.9 kcal/mol, depending on which size-consistency correction is used); and to within 2 kcal/mol for the worst case of NEVPT2 (MAD 0.7 kcal/mol) (see Figure S4, Supporting Information).

### 3.4 Comparison of Several CCSD(T) Protocols

**Figure 10:**
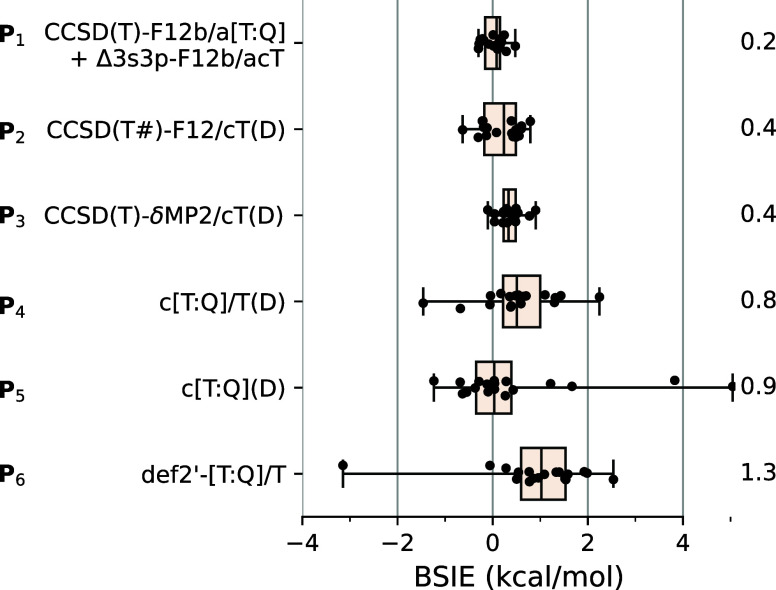
Basis set incompleteness errors (BSIEs, kcal/mol) for the CCSD(T) spin-state energetics of the benchmark set calculated using computational protocols **P**_**1**_**–P**_**6**_ (see text for discussion). Annotated values are MADs.

The above considerations allow us to define several computational protocols to approach the CBS limits of spin-state energetics at the CCSD(T) level. Their performance, in terms of the residual BSIEs over our test set of TM spin-state energetics, is summarized in Figure 10.

The first protocol, **P**_**1**_, is based on eq 9, i.e., the a[T:Q] extrapolation of the CCSD(T)-F12b energies supplemented with the TM outer-core correlation correction calculated using the acT basis set. As already mentioned, **P**_**1**_ is similar to the approach of Peterson with co-workers for small TM molecules and it yields energy differences of excellent accuracy (comparable to the accuracy of our reference CBS limits). Although **P**_**1**_ is computationally very expensive and intractable for larger complexes, we included it in Figure 10 for the sake of comparison. The second protocol, **P**_**2**_, is based on eq 11, i.e., the CCSD(T#)-F12a/cT(D) calculations, and has been extensively discussed in Section 3.2. Despite its simplicity, **P**_**2**_ yields spin-state energetics with the BSIE below 1 kcal/mol and the MAD as small as 0.4 kcal/mol. The third protocol, **P**_**3**_, denoted CCSD(T)-δMP2/cT(D), is a focal-point approach based on eq 15, i.e., exploring a very good BSIE transferability between the CCSD(T) and a computationally cheaper MP2 method. For our test set of spin-state energetics, **P**_**3**_ performs comparably well as **P**_**2**_. Note, however, that we assumed here the exact MP2/CBS limits, whereas further approximations would have to be introduced in applications to larger complexes.

The remaining protocols are based on the CBS extrapolation of conventional CCSD(T) energies. For **P**_**4**_, the extrapolation denoted as c[T:Q]/T(D) is performed only with respect to the TM’s basis set while keeping fixed the ligands basis set: triple-ζ for atoms directly bonded to the TM but only double-ζ for other ligand atoms (see Section 2.4 for the notation of basis sets and extrapolations). For **P**_**5**_, the extrapolation denoted as c[T:Q](D) is performed with respect to the basis set on the TM atom and the ligand atoms directly bonded to it while keeping fixed the double-ζ basis for other ligand atoms. In these protocols, the reduction of the basis set far from the TM atom is made analogously as in **P**_**2**_ and **P**_**3**_, but their BSIEs turn out to be noticeably larger. The maximum BSIE for **P**_**4**_ exceeds 2 kcal/mol in the case of ^4,6^[FeL_2_(Cl)]; for **P**_**5**_, it reaches 5 kcal/mol in the case of .

The last computational protocol, **P**_**6**_, is similar to the basis set extrapolations performed by Pantazis and co-workers in their recent studies of manganese SCO complexes^64^ and oxygen-evolving complex.^101^ The extrapolation denoted def2′-[T:Q]/T was performed with respect to TM’s basis set (triple- and quadruple-ζ) while keeping fixed the triple-ζ basis set on the ligands (reduced to double-ζ on H atoms). We used identical extrapolation formulas as Pantazis and co-workers [(see eqs S7 and S8) in Supporting Information] and employed the Ahlrichs def2 basis sets similar to their choice of ZORA-def2 basis sets.^102^ To comply with the ZORA-def2 basis sets, which are uncontracted, except for the innermost core orbitals,^102^ we analogously uncontracted the original def2 basis sets used in these calculations (denoted def2′). As shown in Figure 10, the BSIEs of **P**_**6**_ are rather disappointing (especially in comparison with similarly constructed **P**_**4**_), with the MAD of 1.3 kcal/mol and maximum deviation of −3.1 kcal/mol for . Even larger BSIEs would be observed for the original def2 basis sets (i.e., without the partial uncontraction made here). The inferior performance of **P**_**6**_ is certainly rooted in the usage of the valence-only basis set (def2) for describing TM outer-core correlation effects.

#### 3.4.1 Comparison of Canonical CCSD(T) with DLPNO–CCSD(T) for MnCp_2_

**Figure 11:**
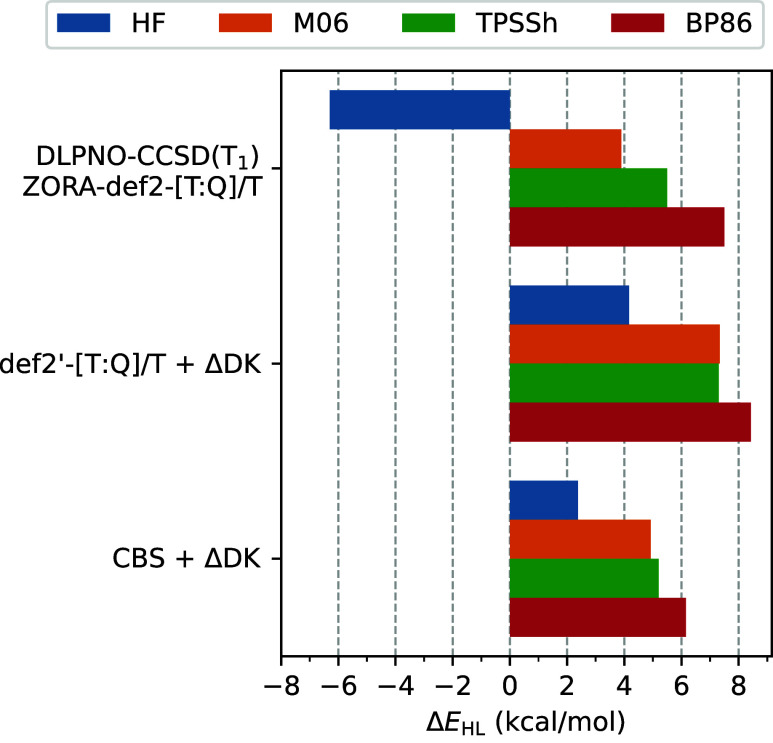
CCSD(T) doublet–sextet splitting for MnCp_2_ with different choices of reference orbitals (HF, M06, TPSSh, and BP86), including scalar-relativistic effects: (a) DLPNO–CCSD(T_1_) results from ref (64), (b) CCSD(T) results extrapolated using modified def2 basis sets analogously as in ref (64), and (c) best estimates of CCSD(T)/CBS limits (see text).

We further note that the sextet–doublet splitting for manganocene (MnCp_2_), one of the complexes studied here, was also investigated by Pantazis with co-workers^64^ at the DLPNO–CCSD(T) level. In Figure 11, we compare their DLPNO–CCSD(T_1_) results (extrapolated to the complete PNO space) with the present canonical CCSD(T) results for several choices of reference orbitals (HF, M06, TPSSh, BP86). To facilitate direct comparison with the results from ref (64), we performed calculations for the same set of geometries and included scalar-relativistic corrections at the Douglas–Kroll level (ΔDK). Moreover, the analogous def2′-[T:Q]/T extrapolation scheme was used as in ref (64), despite its imperfections revealed above (cf. **P6** in Figure 10). For the sake of completeness, Figure 11 also includes refined estimates of the CCSD(T)/CBS limits obtained using the CCSD(T#)-F12a/cT(D) approach, which differ by ∼2 kcal/mol. Further computational details of our calculations for MnCp_2_ can be found in Supporting Information (Section S6).

When comparing the DLPNO–CCSD(T) results from ref (64) and the present CCSD(T) results (def2′-[T:Q]/T + ΔDK), the main difference is rooted in the usage of the DLPNO approximation in the former ones. As clearly shown in Figure 11, the DLPNO–CCSD(T) and canonical CCSD(T) results for MnCp_2_ differ by a modest ∼2 kcal/mol for the choice of BP86 or TPSSh reference orbitals, by ∼3 kcal/mol for the M06 orbitals, and by more than 10 kcal/mol for the HF orbitals. Thus, the discrepancy—which is attributed to the DLPNO truncation error—tends to increase with a growing percentage of admixed exact exchange (EE): none in BP86, 10% in TPSSh, and 25% in M06, to eventually become very large in the case of HF orbitals (formally, 100% of the EE).

A related observation was made in another study of Pantazis with co-workers,^103^ who observed that the DLPNO truncation error for one of their Fe^III^ complexes is greater for HF than for KS orbitals. It is clear that these truncation errors may be reduced by tightening the accuracy thresholds, extrapolating the correlation energy to the limit of a PNO space,^104^ and using the new (T_1_) iterative formulation for the triples correction term.^22,103^ However, eliminating these errors entirely may be difficult (perhaps, more challenging than is currently believed), which is illustrated not only by the presently shown results but also by Reimann and Kaup for the set of five Fe^II^ complexes^25a^ and by Feldt et al. for oxo-Fe^IV^ complexes.^20,105^ In this regard, the case of MnCp_2_ seems to be quite challenging, perhaps due to great covalency of its metal–ligand bond. More systematic studies on the accuracy of the DLPNO approximation (for chemically diverse TM complexes and various choices of reference orbitals) would be thus very interesting to carry out.

The discussed DLPNO truncation error is also presumably the main reason for the pronounced sensitivity of the DLPNO–CCSD(T) energies to the choice of reference orbitals.^64,101,103,106,107^ In canonical CCSD(T) calculations, the effect of reference orbitals is much more limited. For the present case of MnCp_2_, the difference caused by switching from M06 to HF orbitals is only 3 kcal/mol (to be compared with the 10 kcal/mol difference observed with the DLPNO approximation); the difference caused by switching from BP86 to M06 orbitals is only 1.5 kcal/mol (DLPNO: 3.6 kcal/mol). Relatively small differences between canonical CCSD(T) results based on HF and KS orbitals were also observed for other complexes in the present study (cf. Table S16, Supporting Information) as well as in our previous studies.^26,27,66^ This adds an interesting point to the long-standing discussion^20,56,107−112^ on the relative merits of using KS reference orbitals in CC calculations: In the case of DLPNO–CCSD(T) calculations, the usage of KS orbitals may be beneficial to reduce the DLPNO truncation errors (more efficiently than in the case of HF orbitals), but this does not determine whether or not KS orbitals are intrinsically better suited than HF orbitals for obtaining accurate energetics at the CCSD(T) level, leaving this question still open.^9,112^

### 3.5 Illustrative Application: CCSD(T) for Metalloporphyrins

**Figure 12:**
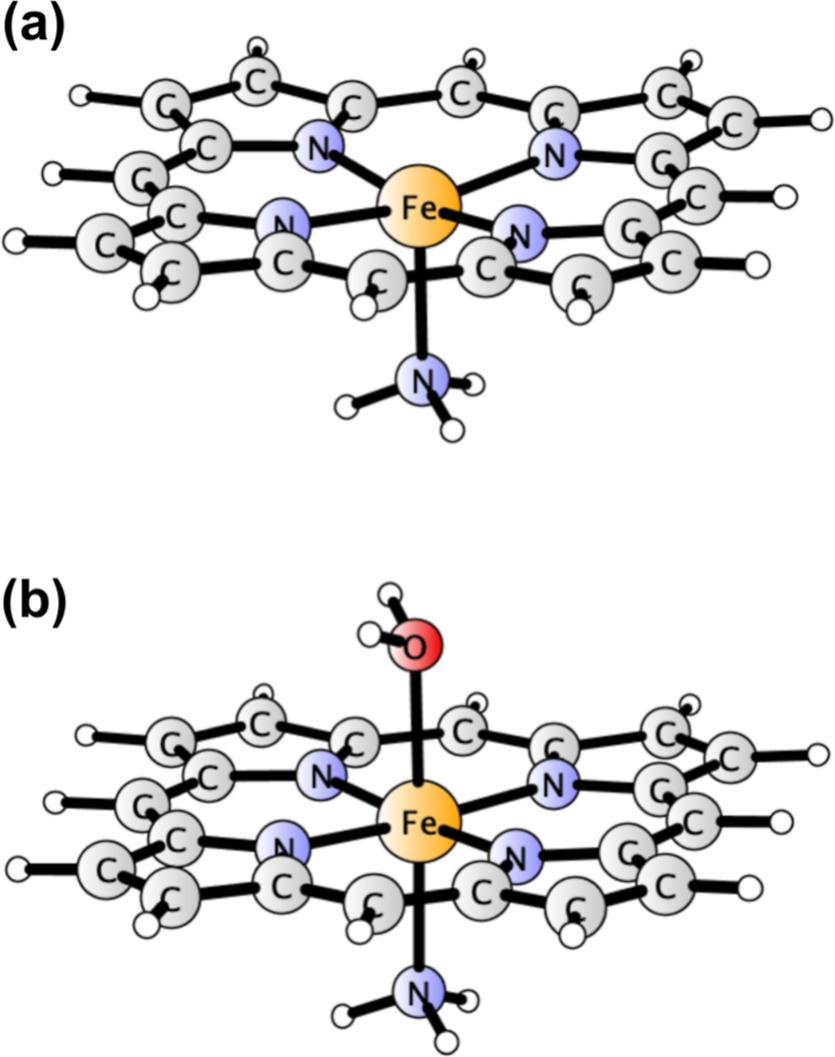
Structures of (a) Fe^II^P(NH_3_) and (b) .

The efficient computational protocol to approximate the CCSD(T)/CBS spin-state energetics using CCSD(T#)-F12a/cT(D) calculations (eq 11), introduced and validated in the present study, can be applied to much larger TM complexes than discussed so far. As an illustration, we apply it to metalloporphyrin complexes: four-coordinate Fe^II^P, five-coordinate Fe^II^P(NH_3_) and , and six-coordinate , where P is porphin (see the structures shown in Figures 5 and 12). These complexes can be regarded as models of ferrous and ferric heme, whose spin-state energetics is of significant interest.^8,17,113−116^ Specifically, NH_3_ is a simplified model of the histidine or imidazole (Im) axial ligands present in active sites of metalloproteins or biomimetic complexes. Thus, Fe^II^P(NH_3_) is a simplified model of Fe^II^P(Im), which in turn is the model of a five-coordinate ferrous heme site in deoxymyoglobin (see ref (17) and references therein). Analogously,  and  are minimal models of the ferric heme site in microperoxidase (see ref (116) and references therein). In the case of Fe^III^, we consider relative energies of the doublet (^2^LS, low-spin) and quartet (^4^IS, intermediate-spin) states with respect to the sextet (^6^HS, high-spin) state and, in the case of Fe^II^, the singlet (^1^LS) and triplet (^3^IS) states with respect to the quintet (^5^HS) state. For Fe^II^P, we do not consider a high-energy singlet state but instead calculate two triplet states lying close in energy: the nondegenerate (^3^IS_1_) and degenerate (^3^IS_2_) ones.^17^ The symmetries, orbital occupancies, and atomic coordinates of the electronic states can be found in Supporting Information. For Fe^II^P, the coordinates are taken from ref (17) (B3LYP-optimized), and for  and , they are taken from ref (116); the geometry of [FeP(NH_3_)] was analogously optimized at the B3LYP-D3(BJ)/def2-TZVP level.

The adiabatic spin-state splittings of the four metalloporphyrins are given in Table 4 (relative to the high spin state).

**Table 4:**
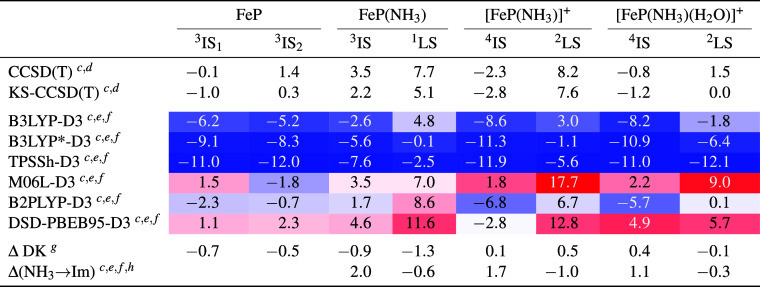
Spin–State Energetics of Studied Metalloporphyrins^a^

a Adiabatic energies in kcal/mol relative to the HS state (^5^HS for Fe^II^ and ^6^HS for Fe^III^ complexes).

b For DFT methods, the cells are color-mapped by deviation from the mean of the CCSD(T) and KS-CCSD(T) results: red for positive and blue for negative deviations.

c Nonrelativistic results.

d Estimated CBS limit (see text).

e Basis set: def2-QZVPP (Fe) and def2-TZVPP (ligands).

f D3 correction with Becke–Johnson damping (or zero damping, in the case of M06L).

g Scalar relativistic correction obtained from the difference between CCSD(T)/cT(D)-DK relativistic and CCSD(T)/cT(D) nonrelativistic results; cT(D)-DK basis set is recontracted for use with the DK Hamiltonian.

h Effect of changing the axial ligand from NH_3_ to Im at the B3LYP-D3(BJ) level.

The first two rows contain nonrelativistic estimates of the CBS limits for CCSD(T) and KS-CCSD(T) with B3LYP orbitals. The CCSD(T)/CBS limits were obtained from the CCSD(T#)-F12a protocol of eq 11, whereas the KS-CCSD(T)/CBS limits were obtained using eq 16 based on the excellent BSIE transferability between the CCSD(T) and KS-CCSD(T) methods (see Section 3.3). The next few rows contain some DFT results (which will be discussed below) and the last two rows contain scalar-relativistic corrections (ΔDK, estimated at the second-order Douglas–Kroll level) and corrections for imperfect representation of the Im axial ligand by ammonia [(Δ(NH_3_ → Im), estimated at the DFT level]. Upon considering these corrections, the CCSD(T) spin-state energetics are qualitatively in agreement with the experimental ground states of similar metalloporphyrins:^8,17,115,116^ the triplet in four-coordinate Fe^II^, the quintet in five-coordinate Fe^II^, and the sextet and quartet state lying very close in energy for Fe^III^ porphyrins.

Due to the improvements made in the present computational protocol and its extensive testing above, the energy differences reported here are to be considered more reliable than earlier results.^17,116^ In particular, for Fe^II^P, the present value of the ^3^IS_1_–^5^HS energy difference is −0.8 kcal/mol (including scalar-relativistic correction), i.e., less negative than the previously reported −2.3 kcal/mol.^17^ With B3LYP orbitals, the energy difference is −1.7 kcal/mol. In view of the very small quintet–triplet splitting, proper interpretation of the experimental (triplet) ground state requires accounting for the crystal packing and vibrational effects.^8,9^ With regard to imidazole-ligated ferrous heme, the present CCSD(T)/CBS estimates (including the scalar-relativistic and NH_3_ → Im corrections) are 4.6 kcal/mol for the ^3^IS–^5^HS and 5.8 kcal/mol for the ^1^LS–^5^HS energy difference. These values agree to within 1.6 kcal/mol with the previously reported estimates (3.0 and 4.6 kcal/mol, respectively), which were based on the calculations for a much more simplified model, FeL_2_(NH_3_).^17^

No quantitative experimental values are available for the discussed energy differences, but in view of the existing benchmark studies,^9,26,27^ it is reasonable to take the present CC results as the reference data for the assessment of DFT methods. The results for some representative functionals are included in Table 4, where the cells are color-mapped by deviation of a given DFT result from the mean of the CCSD(T) and KS-CCSD(T) results. It is immediately seen that commonly used hybrid functionals—B3LYP, B3LYP*, and TPSSh—tend to overstabilize the LS and IS states with respect to the HS state. These effects are relatively minor in Fe^II^ porphyrins but larger in Fe^III^ porphyrins, resulting in a dramatic overstabilization of their ^4^IS state, which was noted already in ref (116). The ^4^IS is predicted to be too low in energy by 5–7 kcal/mol already with the standard B3LYP functional containing 20% of the exact exchange. Hybrid functionals containing lower admixtures of the exact exchange, such as 15% is B3LYP* or 10% in TPSSh—in spite of being often recommended for modeling TM complexes^11,117−119^—perform even worse because they increase the overstabilization error to 9–10 kcal/mol. By contrast, the M06L functional is not biased in favor of the ^4^IS state but tends to overstabilize the HS states. In the particular case of FeP(NH_3_), M06L gives results very close to the CCSD(T) ones, but the agreement is not equally good for other complexes. As a matter of fact, none of the tested functionals provide perfect agreement with the CCSD(T) or KS-CCSD(T) spin-state energetics in the case of all four metalloporphyrins simultaneously, which is not surprising to us,^8,17,26^ but the two double-hybrid functionals (B2PLYP and DSD-PBEB95) perform promisingly well.

## 4 Conclusions

We analyzed the basis set convergence in conventional and explicitly correlated CCSD(T) calculations of TM spin-state energetics for the benchmark set of 18 energy differences in 13 chemically diverse complexes. Based on these considerations, we developed a computationally efficient protocol to approximate the CCSD(T)/CBS limits of TM spin-state energetics using explicitly correlated CCSD(T#)-F12a calculations with a relatively small basis set (for the TM atom: cc-pwCVTZ; for ligand atoms: cc-pVTZ, if bonded to the TM atom or cc-pVDZ otherwise). In order to increase the accuracy of the perturbative triples contribution, a modified scaling of this term was introduced, denoted (T#). The proposed computational protocol was shown to reproduce the reference CCSD(T)/CBS spin-state energetics from the benchmark set to within the chemical accuracy (mean deviation of 0.2, MAD of 0.4, and maximum deviation of 0.8 kcal/mol) with a modest computational effort in comparison with traditional extrapolation approaches or more accurate (but also more expensive) F12b-based protocols described in the literature.

The proposed efficient protocol is tailored to calculations of spin-state energetics in first-row TM complexes. It benefits from certain features in their electronic structures—such as the locality of changes caused by the change of TM’s spin state and considerable overlap between the TM and ligands basis functions, leading to the observed unimportance of diffuse functions—which result in the possibility of making considerable computational savings with only a minor decrease of the accuracy. One should not expect this protocol to be equally accurate in general thermochemical applications (e.g., atomization energies) or for complexes with noninnocent ligands. We foresee possible applications in the calculations of ligand binding energies and redox potentials of TM complexes, but these would require further benchmarking on appropriate model systems.

The robustness of our computational protocol was illustrated by its application to metalloporphyrins being simplified models of Fe^II^ and Fe^III^ heme groups, for which refined spin-state energetics at the CCSD(T) level were obtained (with the aim of improving previous estimates for similar models^17,116^). In practice, the proposed protocol is applicable to mononuclear TM complexes sized up to about 50 atoms, including realistic models of many SCO complexes characterized experimentally. Performing calculations on even larger TM complexes would require combining it with local correlation treatments, for example, the PNO^62^ or DLPNO^63^ approximations. However, with regard to the accuracy of these approaches for spin-state energies, one should be aware of the truncation errors, which may be still significant in practice. The problem was analyzed for one of the studied complexes by comparing the DLPNO–CCSD(T) results taken from the literature with the present canonical CCSD(T) results. It was revealed that the DLPNO truncation error is sensitive to the choice of orbitals in the reference determinant and the error is much greater for HF orbitals than for KS orbitals. This is presumably one of the main reasons of the claimed advantages of using KS orbitals, often proffered in the literature. We thus believe that it still remains an open question which choice of orbitals (HF or KS) is more appropriate for obtaining accurate spin-state energetics at the CCSD(T) level. As local correlation approximations may introduce truncation errors that depend on the choice of orbitals, the question will be best answered through canonical CCSD(T) calculations, such as those performed in this study.

Another important finding in this article is a practical demonstration of a very good transferability of the BSIEs between CCSD(T) and other WFT methods, such as CASPT2, MRCI+Q, or even MP2. The observed BSIE transferability, on one hand, motivates focal-point approximations in which the BSIE is additively corrected for using a computationally cheap method, for example, MP2. In this regard, MP2 works, despite its well-known shortcomings for TM complexes, because it is a good predictor of the BSIE. On the other hand, the BSIE transferability greatly simplifies the construction of benchmark studies based on experimental reference data: unless a very high precision of the calculated energies is required, it is legitimate to compare the results of different methods in a finite basis set and apply common correction to the CBS limit (instead of independently extrapolating the result of each method to the CBS limit).

## Supporting Information Available

Additional data, complete refs (67,69, and 76), and sample input file for CCSD(T#)-F12a/cT(D) calculations (PDF)
Total energies (XLSX)
Cartesian coordinates and orbital occupations (TXT)
