# Supplementary material for: Approaching the Complete Basis Set Limit for Spin-State Energetics of Mononuclear First-Row Transition Metal Complexes
Source: J Chem Theory Comput. 2024 Apr 4;20(8):3199–217. doi: 10.1021/acs.jctc.4c00092 (PMC11044276; doi:10.1021/acs.jctc.4c00092)
Supplement: Supplementary file 1 — ct4c00092_si_001.pdf [file ct4c00092_si_001.pdf]

Supporting Information: Approaching the Complete Basis Set Limit for Spin–State Energetics of Mononuclear First-Row Transition Metal Complexes

Gabriela Drabik†,‡ and Mariusz Radoń*,‡

†*Jagiellonian University, Doctoral School of Exact and Natural Sciences, 30-348 Kraków, Poland*

‡*Jagiellonian University, Faculty of Chemistry, 30-387 Kraków, Poland*

E-mail: mradon@chemia.uj.edu.pl

Phone: +48 12 6862489

Contents

List of Tables S-3

List of Figures S-4

1 Description of Active Spaces S-5

2 Auxiliary Basis Sets for F12 Calculations S-7

3 Comparison of Alternative CBS Extrapolations S-8

4 Detailed CCSD(T) and CCSD(T)-F12 Results S-14

5 Results of Other Methods, BSIE Transferability S-25

6 Additional Calculations for $MnCp_2$ S-30

7 Sample Input file for CCSD(T#)-F12a Calculations S-32

8 Complete References 67, 69, and 76 S-34

References S-34

List of Tables

S1 Description of Active Spaces in Multiconfigurational Calculations. S-5

S2 Auxiliary Basis Sets for F12 Calculations S-7

S3 Comparison of Alternative CBS Extrapolations, CCSD(T)/ac[Q:5]/Q S-10

S4 Comparison of Alternative CBS Extrapolations, CASPT2/ac[Q:5]/Q S-11

S5 Comparison of Alternative CBS Extrapolations, FC-CCSD/a[T:Q] S-12

S6 CCSD(T) Spin–State Energetics from ac[Q:5]/Q and ac[T:Q] Extrapolations S-13

S7 BSIE of the FC-CCSD and FC-CCSD-F12a/b Spin–State Energetics S-14

S8 BSIE of the FC-(T) Contribution to Spin–State Energetics S-15

S9 BSIE of the Δ3s3p Contribution to Spin–State Energetics S-16

S10 BSIE of the FC-CCSD-F12a Spin–State Energetics Using Triple-ζ Basis Sets . . . S-18

S11 Effect of Diffuse Functions in Conventional and F12 Calculations S-20

S12 Comparison of /T and /cT Results at FC-CCSD(-F12) Level S-21

S13 BSIE of the FC-(T), (T*), (T#) Terms in F12a Calculations S-22

S14 BSIE of the Δ3s3p Term in CCSD(T)-F12a/cT(D) Calculations S-23

S15 BSIE of CCSD(T)-F12a/cT(D) Energetics with Different Variants of (T) Term . . . S-24

S16 Spin–State Energetics from Various Methods, CBS Limits S-26

S17 Spin–State Energetics from Various Methods, cT(D) Basis Set S-27

S18 Differential BSIEs Using cT(D) Basis Set S-28

S19 Non-relativistic and Scalar-Relativistic Results for Different Structures of $MnCp_2$. S-31

S20 DLPNO-CCSD(T0) Results for $MnCp_2$ S-31

S21 Results of Sample CCSD(T#)-F12a Calculations S-33

List of Figures

S1 Timings of selected conventional and explicitly correlated CCSD(T) calculations of the quintet–singlet splitting for $[Fe(NCH)_6]^{2+}$ S-17

S2 Timings of selected FC-CCSD-F12 and CCSD(T)-F12 calculations of the quintet–singlet splitting for $[Fe(NCH)_6]^{2+}$ S-19

S3 BSIE of the CCSD(T,α)-F12a/cT(D) spin–state energetics as function of α S-21

S4 BSIEs for approximate CBS limits obtained using eq. (16), main article S-25

S5 Heat-map of the Pearson's correlation matrix between the BSIEs of all considered WFT methods calculated for the benchmark set of spin–state energetics using the cT(D) basis set. S-29

1 Description of Active Spaces

Table S1 gives detailed description of the active spaces used in multiconfigurational calculations.

Table S1: Description of Active Spaces in Multiconfigurational Calculations.

complex	active space[a]	active orbitals[b]	core fixing[c]
$[Fe(NCH)_6]^{2+}$	(10,12)	Fe 3d (5), 4d (5), $\sigma_{Fe–N}$ (2)	3s
$[Fe(NCH)_6]^{3+}$	(9,12)	Fe 3d (5), 4d (5), $\sigma_{Fe–N}$ (2)	
$[Co(NCH)_6]^{2+}$	(11,11/12)[d]	Co 3d (5), 4d (4/5)[d], $\sigma_{Co–N}$ (2)	3s
$[Fe(CNH)_6]^{2+}$	(10,12)	Fe 3d (5), 4d (5), $\sigma_{Fe–C}$ (2)	
$[Fe(CNH)_6]^{3+}$	(9,12)	Fe 3d (5), 4d (5), $\sigma_{Fe–C}$ (2)	
$[FeO(NH_3)_5]^{2+}$	(12,15/16)[d]	Fe 3d (5), 4d (3/4)[d], O 2p (3), 3p (3), $\sigma_{Fe–N}$ (1)	
$[Fe(H_2O)_6]^{2+}$	(10,10/12)[d]	Fe 3d (5), 4d (3/5)[d], $\sigma_{Fe–O}$ (2)	3s
$[Fe(H_2O)_6]^{3+}$	(9,12)	Fe 3d (5), 4d (5), $\sigma_{Fe–O}$ (2)	
$FeCp_2$	(10,12)	Fe 3d (5), 4d (5), Fe–Cp (2)	
$MnCp_2$	(9,12)	Mn 3d (5), 4d (5), Mn–Cp (2)	
FeL_2	(8,10/11/11)[d]	Fe 3d (5), 4d (4/5/5)[d], $\sigma_{Fe–N}$ (1)	
$FeL_2(Cl)$	(9,12)	Fe 3d (5), 4d (5), $\sigma_{Fe–N}$ (1), $\sigma_{Fe–Cl}$ (1)	
$FeL_2(NH_3)$	(10,10/11/12)[d]	Fe 3d (5), 4d (3/4/5)[d], $\sigma_{Fe–N}$ (2)	3s3p

[a]Notation (N_e, N_o) is used, where N_e is the number of active electrons, N_o the number of active orbitals. [b]Number of orbitals of a given kind in parenthesis. [c]Core-fixing procedure (described in the main article) applied in some cases to either 3s or 3s3p orbitals of the TM atom. [d]Slash-separated numbers apply to different spin states, in the order from lower- to higher-spin.

Number of active double–shell orbitals. It was attempted to make the active space compatible for all spin states of a given complex. In some cases, it turned out necessary to make the number of correlating 4d orbitals different in different spin states with the following rule: the $4d_i$ orbital was made active only if the corresponding $3d_i$ orbital is occupied in the leading configuration. (For lower-spin states, very weakly occupied 4d orbitals are not needed to describe the double–shell effect; if they are included in the active space, they undergo uncontrolled rotations, leading to incorrect character of the active orbitals obtained after formally converging the CASSCF calculations.) For example, in the case $Fe^{II}L_2(NH_3)$ complex, the number of active orbitals is 12 for the quintet (i.e., all five 4d orbitals are active), 11 for the triplet (i.e., only four 4d orbitls are active, as one of the 3d orbitals is no longer significantly occupied), and 10 for the singlet (i.e., only three 4d orbitals are active as two of the 3d orbitals are no longer significantly occupied).

Core fixing procedure. For some complexes it turned out necessary to apply a core-fixing procedure, similar to the one used for $[Fe(NCH)_6]^{2+}$ by Pierloot with co-workers,[S4] in order to prevent the 3s or 3s3p outer-core orbitals from entering the active space (and replacing the desired metal–ligand bonding orbitals). Wherever necessary (see Table S1 for details), the CASSCF optimization of orbitals was performed in the following steps, starting from the initial guess taken from a smaller active space, where only the TM 3d and 4d orbitals were active. In step #1, CASSCF calculations were performed in which 3s or 3s3p orbitals were kept fixed. In step #2, the metal–ligand bonding orbitals were kept fixed. The two-step procedure was iterated until the total CASSCF energies obtained after steps #1 and 2 were identical (just a few iterations were enough). In order to keep fixed selected orbitals during CASSCF orbital optimization, the `supersymmetry` keyword was used in OpenMolcas, whereas the `freeze` keyword was used in Molpro. It was checked that identical CASSCF energies are obtained in different programs.

2 Auxiliary Basis Sets for F12 Calculations

Table S2: Auxiliary Basis Sets for F12 Calculations

orbital basis	auxiliary basis	transition metal	other atoms
aT, aQ, T, Q	df_basis[a]	aug-cc-pV*n*Z/mp2fit<br>(*n* =T,[S5] Q[S6])[d,e]	aug-cc-pV*n*Z/mp2fit[S7]<br>(*n* =T, Q)[d,f]
	df_basis_exch[b]	def2-QZVPP/jkfit[S8]	cc-pV*n*Z/jkfit[S9]<br>(*n* =T, Q)[d,f]
	ri_basis[c]	aug-cc-pV*n*Z/mp2fit<br>(*n* =T,[S5] Q[S6])[d,e]	aug-cc-pV*n*Z/optri[S10]<br>(*n* =T, Q)[d,f]
cT, cT(D), cT/D	df_basis[a]	aug-cc-pVTZ/mp2fit[S5]	aug-cc-pV*n*Z/mp2fit[S7]<br>(*n* =T, D)[d,f]
	df_basis_exch[b]	def2-TZVPP/jkfit[S8]	aug-cc-pV*n*Z/jkfit[S9]<br>(*n* =T, D)[d,f]
	ri_basis[c]	def2-TZVPP/jkfit[S8]	aug-cc-pV*n*Z/optri[S10]<br>(*n* =T, D)[d,f]

[a]Basis set for density fitting. [b]Density fitting basis for the exchange and Fock operators. [c]Basis set for the resolution of the identity. [d]The choice of *n* matches the orbital basis set. [e]The *k*-functions were excluded from the aug-cc-pVQZ/mp2fit basis set for TM atoms, as in ref S6. [f]For chlorine, (aug-)cc-pV(T+*n*)Z (and corresponding auxiliary basis) is used instead of (aug-)cc-pV*n*Z.

3 Comparison of Alternative CBS Extrapolations

As explained in the main article, the reference CBS limits used in this work were obtained from the a[Q:5]/Q extrapolation in which the correlation energy was extrapolated using the formula

$$E_{\mathrm{corr}}(\mathrm{CBS}) = \frac{(m+\frac{3}{2})^4 E_{\mathrm{ref}}(m) - (n+\frac{3}{2})^4 E_{\mathrm{ref}}(n)}{(m+\frac{3}{2})^4 - (n+\frac{3}{2})^4}, \tag{S.1}$$

whereas the reference energy (HF, CASSCF) was extrapolated using the formula

$$E_{\mathrm{ref}}(\mathrm{CBS}) = \frac{e^{\alpha m} E_{\mathrm{ref}}(m) - e^{\alpha n} E_{\mathrm{ref}}(n)}{e^{\alpha m} - e^{\alpha n}}, \quad \alpha = 1.63 \tag{S.2}$$

where $m = 4$ for the acQ basis and $n = 5$ for the ac5/Q basis. Comparison with some alternative CBS extrapolation schemes can be found in Tables S3 and S4 for CCSD(T) and CASPT2 results, respectively. The alternative extrapolation scheme tested here are following:

- the E_{ref} taken from the larger basis set, i.e. formally

$$E_{\mathrm{ref}}(\mathrm{CBS}) = E_{\mathrm{ref}}\left(\max(m, n)\right); \tag{S.3}$$

  the E_{corr} extrapolated using eq. (S.1);

- the E_{ref} extrapolated using eq. (S.2), the E_{corr} extrapolated using eq. (S.4);

$$E_{\mathrm{corr}}(\mathrm{CBS}) = \frac{m^3 E_{\mathrm{corr}}(m) - n^3 E_{\mathrm{corr}}(n)}{m^3 - n^3} \tag{S.4}$$

- the total energy ($E_{\mathrm{ref}} + E_{\mathrm{corr}}$) extrapolated using eq. (S.1).

As can be seen, the results of the ac[Q:5]/Q extrapolation are almost insensitive (to within 0.1 kcal/mol) to the choice of the extrapolation scheme.

In addition, for the analysis of FC-CCSD/a[T:Q] energies, presented later in Table S5, we considered the following two extrapolation approaches making use of the parameters empirically

optimized for the given pair of basis sets:

- Schwenke type[S11] extrapolations for HF and correlation energy

$$E_{\mathrm{HF}}(\mathrm{CBS}) = E_{\mathrm{HF}}(n) + F^{\mathrm{HF}}_{n,n+1}\left(\{E_{\mathrm{HF}}(n+1) - E_{\mathrm{HF}}(n)\right\}, \tag{S.5}$$

$$E_{\mathrm{corr}}(\mathrm{CBS}) = E_{\mathrm{corr}}(n) + F^{\mathrm{corr}}_{n,n+1}\left\{E_{\mathrm{corr}}(n+1) - E_{\mathrm{corr}}(n)\right\}, \tag{S.6}$$

  using Schwenke coefficients ($F^{\mathrm{HF}}_{n,n+1}$, $F^{\mathrm{corr}}_{n,n+1}$) tabulated in ref S11 for a given pair of basis sets with cardinal numbers n and $n+1$. For the pair of basis sets aT:aQ, we used $F^{\mathrm{HF}}_{3,4} = 1.2940531$ for the HF energy and $F^{\mathrm{corr}}_{3,4} = 1.7001115$ for the CCSD correlation energy.

- Truhlar type[S12] extrapolation of correlation energy

$$E_{\mathrm{corr}}(\mathrm{CBS}) = \frac{m^{\beta}E_{\mathrm{corr}}(m) - n^{\beta}E_{\mathrm{corr}}(n)}{m^{\beta} - n^{\beta}} \tag{S.7}$$

  combined with the extrapolation of the HF energy

$$E_{\mathrm{ref}}(\mathrm{CBS}) = \frac{e^{\alpha\sqrt{m}}E_{\mathrm{ref}}(m) - e^{\alpha\sqrt{n}}E_{\mathrm{ref}}(n)}{e^{\alpha\sqrt{m}} - e^{\alpha\sqrt{n}}}, \tag{S.8}$$

  whose functional form originates in the study of Klopper and Kutzelnigg[S13] and is also similar to the Karton–Martin type extrapolation.[S14] In eq. (S.7) and (S.8), α and β are parameters optimized for a given pair of basis sets. For the basis sets aT:aQ, we used the values $\alpha = 5.79$ and $\beta = 3.05$ provided by Neese and Valeev in ref S15.

Note that eqs (S.7) and (S.8) with α and β specific to a given pair of basis sets are functionally equivalent[S11,S16] to eqs (S.5) and (S.6) with certain Schwenke coefficients.

Table S3: Comparison of Alternative CBS Extrapolations, CCSD(T)/ac[Q:5]/Q Energies. [a,b]

	E_{ref}: eq. (S.3) E_{corr}: eq. (S.1)	E_{ref}: eq. (S.2) E_{corr}: eq. (S.4)	E_{tot}: eq. (S.1)
^{1,5}[Fe(NCH)$_6$]$^{2+}$	−0.02	−0.00	0.06
^{2,6}[Fe(NCH)$_6$]$^{3+}$	−0.02	−0.00	0.06
^{2,4}[Co(NCH)$_6$]$^{2+}$	−0.01	−0.00	0.03
^{1,5}[Fe(CNH)$_6$]$^{2+}$	−0.02	−0.00	0.06
^{2,6}[Fe(CNH)$_6$]$^{3+}$	−0.02	−0.00	0.06
^{3,5}[FeO(NH$_3$)$_5$]$^{2+}$	−0.01	−0.00	0.03
^{1,5}[Fe(H$_2$O)$_6$]$^{2+}$	−0.02	−0.00	0.07
^{4,6}[Fe(H$_2$O)$_6$]$^{3+}$ (ve)	−0.01	−0.00	0.03
^{1,3}[FeCp$_2$]	−0.00	−0.00	0.00
^{1,3}[FeCp$_2$] (ve)	−0.00	−0.00	0.01
^{2,6}[MnCp$_2$]	−0.02	−0.00	0.06
^{1,5}[FeL$_2$]	−0.02	−0.00	0.07
^{3,5}[FeL$_2$] (1)	−0.01	−0.00	0.03
^{3,5}[FeL$_2$] (2)	−0.01	−0.00	0.04
^{2,6}[FeL$_2$Cl]	−0.02	−0.00	0.07
^{4,6}[FeL$_2$Cl]	−0.01	−0.00	0.03
^{1,5}[FeL$_2$NH$_3$]	−0.02	−0.00	0.07
^{3,5}[FeL$_2$NH$_3$]	−0.01	−0.00	0.04
MAD [c]	0.01	0.00	0.05
|Max| [d]	0.02	0.00	0.07

[a]All values in kcal/mol, relative to the reference CBS limits ac[Q:5]/Q of Table 3 (main article), where the correlation energy (E_{corr}) was extrapolated using eq. (S.1), the reference energy (E_{ref}, i.e. HF energy) was extrapolated using eq. (S.2). [b]Including contribution due to correlating the TM 3s3p electrons. [c]Mean absolute deviation. [d]Maximum absolute deviation.

Table S4: Comparison of Alternative CBS Extrapolations, CASPT2/ac[Q:5]/Q Energies. [a,b]

	E_{ref}: eq. (S.3) E_{corr}: eq. (S.1)	E_{ref}: eq. (S.2) E_{corr}: eq. (S.4)	E_{tot}: eq. (S.1)
^{1,5}[Fe(NCH)$_6$]$^{2+}$	−0.02	−0.00	0.06
^{2,6}[Fe(NCH)$_6$]$^{3+}$	−0.02	−0.00	0.06
^{2,4}[Co(NCH)$_6$]$^{2+}$	−0.01	−0.00	0.04
^{1,5}[Fe(CNH)$_6$]$^{2+}$	−0.01	−0.00	0.04
^{2,6}[Fe(CNH)$_6$]$^{3+}$	−0.02	−0.00	0.05
^{3,5}[FeO(NH$_3$)$_5$]$^{2+}$	−0.01	−0.00	0.04
^{1,5}[Fe(H$_2$O)$_6$]$^{2+}$	−0.02	−0.00	0.08
^{4,6}[Fe(H$_2$O)$_6$]$^{3+}$ (ve)	−0.00	−0.00	0.01
^{1,3}[FeCp$_2$]	−0.00	−0.00	0.00
^{1,3}[FeCp$_2$] (ve)	−0.00	−0.00	0.01
^{2,6}[MnCp$_2$]	−0.01	−0.00	0.04
^{1,5}[FeL$_2$]	−0.02	−0.00	0.07
^{3,5}[FeL$_2$] (1)	−0.01	−0.00	0.03
^{3,5}[FeL$_2$] (2)	−0.01	−0.00	0.03
^{2,6}[FeL$_2$Cl]	−0.02	−0.00	0.06
^{4,6}[FeL$_2$Cl]	−0.01	−0.00	0.03
^{1,5}[FeL$_2$NH$_3$]	−0.02	−0.00	0.06
^{3,5}[FeL$_2$NH$_3$]	−0.01	−0.00	0.03
MAD [c]	0.01	0.00	0.04
|Max| [d]	0.02	0.00	0.08

[a]All values in kcal/mol, relative to the reference CBS limits ac[Q:5]/Q of Table **??** (main article) where the correlation energy (E_{corr}) was extrapolated using eq. (S.1), the reference energy (E_{ref}, i.e. CASSCF energy) was extrapolated using eq. (S.2). [b]Including contribution due to correlating the TM 3s3p electrons. [c]Mean absolute deviation. [d]Maximum absolute deviation.

Table S5: Comparison of Alternative CBS Extrapolations, FC-CCSD/a[T:Q] Energies. [a,b]

	E_{HF}: eq. (S.2) E_{corr}: eq. (S.1) [c]	E_{HF}: eq. (S.3) E_{corr}: eq. (S.1)	E_{HF}: eq. (S.2) E_{corr}: eq. (S.4)	E_{HF}: eq. (S.3) E_{corr}: eq. (S.4)	E_{HF}: eq. (S.5) E_{corr}: eq. (S.6)[d]	E_{HF}: eq. (S.8) E_{corr}: eq. (S.7)[e]
$^{1,5}[Fe(NCH)_6]^{2+}$	−0.6	−0.6	−0.7	−0.7	−0.8	−0.8
$^{2,6}[Fe(NCH)_6]^{3+}$	−1.2	−1.3	−1.4	−1.4	−1.5	−1.4
$^{2,4}[Co(NCH)_6]^{2+}$	−0.2	−0.2	−0.3	−0.3	−0.4	−0.3
$^{1,5}[Fe(CNH)_6]^{2+}$	−0.6	−0.6	−0.8	−0.8	−0.8	−0.8
$^{2,6}[Fe(CNH)_6]^{3+}$	−1.2	−1.2	−1.3	−1.3	−1.4	−1.3
$^{3,5}[FeO(NH_3)_5]^{2+}$	−0.5	−0.5	−0.6	−0.6	−0.6	−0.6
$^{1,5}[Fe(H_2O)_6]^{2+}$	−1.0	−1.1	−1.1	−1.2	−1.1	−1.1
$^{4,6}[Fe(H_2O)_6]^{3+}$ (ve)	−0.9	−0.9	−0.9	−1.0	−1.0	−0.9
$^{1,3}[FeCp_2]$	−0.3	−0.3	−0.3	−0.4	−0.3	−0.3
$^{1,3}[FeCp_2]$ (ve)	−0.2	−0.3	−0.2	−0.3	−0.2	−0.2
$^{2,6}[MnCp_2]$	−1.3	−1.4	−1.4	−1.5	−1.5	−1.4
$^{1,5}[FeL_2]$	−0.8	−0.8	−0.9	−0.9	−0.9	−0.9
$^{3,5}[FeL_2]$ (1)	−0.5	−0.6	−0.6	−0.6	−0.6	−0.6
$^{3,5}[FeL_2]$ (2)	−0.5	−0.5	−0.6	−0.6	−0.6	−0.6
$^{2,6}[FeL_2Cl]$	−1.4	−1.4	−1.5	−1.5	−1.5	−1.5
$^{4,6}[FeL_2Cl]$	−0.8	−0.8	−0.9	−0.9	−0.9	−0.9
$^{1,5}[FeL_2NH_3]$	−0.8	−0.8	−0.9	−0.9	−0.9	−0.9
$^{3,5}[FeL_2NH_3]$	−0.5	−0.5	−0.6	−0.6	−0.6	−0.6
|Max| [f]	1.4	1.4	1.5	1.5	1.5	1.5
MAD [g]	0.7	0.8	0.8	0.9	0.9	0.9

[a]BSIE values in kcal/mol, with respect to the corresponding reference CBS limits from Table 3, main article. [b]Only valence electrons correlated (TM 3s3p in the frozen core, FC). [c]The default extrapolation scheme. [d]Schwenke-type. [e]Truhlar-type. [f]Maximum absolute deviation. [g]Mean absolute deviation.

Table S6: Comparison of CCSD(T) Spin–State Energetics from the ac[Q:5]/Q and ac[T:Q] Extrapolations.[a,b]

	ac[Q:5]/Q	ac[T:Q]
^{1,5}[Fe(NCH)$_6$]$^{2+}$	−7.3	−7.4
^{2,6}[Fe(NCH)$_6$]$^{3+}$	−19.0	−19.9
^{2,4}[Co(NCH)$_6$]$^{2+}$	−11.4	−11.5
^{1,5}[Fe(CNH)$_6$]$^{2+}$	48.7	48.4
^{2,6}[Fe(CNH)$_6$]$^{3+}$	27.5	26.5
^{3,5}[FeO(NH$_3$)$_5$]$^{2+}$	−0.4	−0.6
^{1,5}[Fe(H$_2$O)$_6$]$^{2+}$	−43.9	−44.8
^{4,6}[Fe(H$_2$O)$_6$]$^{3+}$ (ve)	−48.4	−49.1
^{1,3}[FeCp$_2$]	34.3	34.2
^{1,3}[FeCp$_2$] (ve)	48.6	48.5
^{2,6}[MnCp$_2$]	−0.4	−1.4
^{1,5}[FeL$_2$]	−31.1	−31.6
^{3,5}[FeL$_2$] (1)	4.2	3.9
^{3,5}[FeL$_2$] (2)	7.2	6.9
^{2,6}[FeL$_2$Cl]	−7.5	−8.6
^{4,6}[FeL$_2$Cl]	−1.8	−2.5
^{1,5}[FeL$_2$NH$_3$]	−11.9	−12.3
^{3,5}[FeL$_2$NH$_3$]	1.3	1.0

[a]Energy differences in kcal/mol. [b]Using eq. (S.1) for extrapolation of the correlation energy, eq. (S.2) for HF energy.

4 Detailed CCSD(T) and CCSD(T)-F12 Results

Table S7: BSIE of the FC-CCSD and FC-CCSD-F12a/b Spin–State Energetics. [a,b,c]

	FC-CCSD			FC-CCSD-F12a		FC-CCSD-F12b		
	aT	aQ	a[T:Q][d]	aT	aQ	aT	aQ	a[T:Q][e]
^{1,5}[Fe(NCH)$_6$]$^{2+}$	−3.8	−2.1	−0.6	−1.3	0.2	−2.6	−0.4	0.4
^{2,6}[Fe(NCH)$_6$]$^{3+}$	−4.9	−2.9	−1.2	−2.1	−0.1	−3.6	−0.9	0.1
^{2,4}[Co(NCH)$_6$]$^{2+}$	−2.1	−1.1	−0.2	−0.8	0.0	−1.4	−0.3	0.2
^{1,5}[Fe(CNH)$_6$]$^{2+}$	−3.5	−1.9	−0.6	−0.8	0.5	−2.1	−0.2	0.5
^{2,6}[Fe(CNH)$_6$]$^{3+}$	−4.8	−2.8	−1.2	−1.9	0.0	−3.6	−0.9	0.2
^{3,5}[FeO(NH$_3$)$_5$]$^{2+}$	−2.4	−1.4	−0.5	−0.6	−0.0	−1.0	−0.2	0.1
^{1,5}[Fe(H$_2$O)$_6$]$^{2+}$	−2.6	−1.6	−1.0	−1.1	0.3	−1.9	−0.2	0.4
^{4,6}[Fe(H$_2$O)$_6$]$^{3+}$ (ve)	−2.6	−1.6	−0.9	−1.3	−0.1	−2.1	−0.6	0.0
^{1,3}[FeCp$_2$]	−1.0	−0.5	−0.3	0.2	0.2	−0.4	−0.1	−0.0
^{1,3}[FeCp$_2$] (ve)	−0.4	−0.2	−0.2	0.2	0.3	−0.3	−0.0	0.1
^{2,6}[MnCp$_2$]	−3.8	−2.4	−1.3	−1.0	0.2	−2.7	−0.7	0.0
^{1,5}[FeL$_2$]	−3.8	−2.0	−0.8	−1.2	0.2	−2.1	−0.3	0.4
^{3,5}[FeL$_2$] (1)	−2.4	−1.3	−0.5	−0.8	0.1	−1.4	−0.3	0.1
^{3,5}[FeL$_2$] (2)	−2.5	−1.4	−0.5	−0.7	0.1	−1.4	−0.3	0.2
^{2,6}[FeL$_2$Cl]	−4.0	−2.5	−1.4	−1.6	−0.1	−3.4	−1.0	−0.0
^{4,6}[FeL$_2$Cl]	−2.5	−1.6	−0.8	−1.3	−0.2	−2.4	−0.8	−0.1
^{1,5}[FeL$_2$NH$_3$]	−3.5	−1.9	−0.8	−1.1	0.2	−2.2	−0.4	0.3
^{3,5}[FeL$_2$NH$_3$]	−2.1	−1.2	−0.5	−0.6	0.1	−1.4	−0.3	0.1
|Max| [f]	4.9	2.9	1.4	2.1	0.5	3.6	1.0	0.5
MSD [g]	−2.9	−1.7	−0.7	−1.0	0.1	−2.0	−0.4	0.2
MAD [h]	2.9	1.7	0.7	1.0	0.2	2.0	0.4	0.2

[a]BSIE values in kcal/mol, with respect to the corresponding reference CBS limits from Table 3, main article. [b]Only valence electrons correlated (TM 3s3p in the frozen core, FC). [c]This table contains numeric data corresponding to FC-CCSD-related boxplots shown in Figure 2, main article. [d]CBS extrapolation using eq. (2) for correlation energy and eq. (3) for reference energy. [e]Schwenke-style CBS extrapolation.[S17] [f]Maximum absolute deviation. [g]Mean signed deviation. [h]Mean absolute deviation.

Table S8: BSIE of the FC-(T) Contribution to Spin–State Energetics. [a,b,c]

	FC-(T)			FC-(T)-F12			FC-(T*)-F12[d]		
	aT	aQ	a[T:Q][e]	aT	aQ	a[T:Q][f]	aT	aQ	a[T:Q][f]
^{1,5}[Fe(NCH)$_6$]$^{2+}$	−0.6	−0.2	0.1	−0.8	−0.3	0.0	0.1	0.2	0.2
^{2,6}[Fe(NCH)$_6$]$^{3+}$	−0.4	−0.2	0.1	−0.7	−0.3	0.0	0.2	0.2	0.2
^{2,4}[Co(NCH)$_6$]$^{2+}$	−0.3	−0.1	0.1	−0.4	−0.2	0.0	−0.0	0.1	0.1
^{1,5}[Fe(CNH)$_6$]$^{2+}$	−0.9	−0.3	0.2	−1.4	−0.5	0.0	0.1	0.2	0.3
^{2,6}[Fe(CNH)$_6$]$^{3+}$	−0.6	−0.2	0.2	−1.1	−0.4	0.1	0.1	0.2	0.3
^{3,5}[FeO(NH$_3$)$_5$]$^{2+}$	−0.1	0.0	0.1	−0.3	−0.1	0.1	0.0	0.1	0.2
^{1,5}[Fe(H$_2$O)$_6$]$^{2+}$	−0.1	−0.1	−0.0	−0.3	−0.1	−0.1	0.0	0.0	0.1
^{4,6}[Fe(H$_2$O)$_6$]$^{3+}$ (ve)	−0.1	−0.1	0.0	−0.2	−0.1	−0.0	0.2	0.1	0.1
^{1,3}[FeCp$_2$]	−0.5	−0.2	0.1	−0.7	−0.3	0.0	−0.2	−0.0	0.1
^{1,3}[FeCp$_2$] (ve)	−0.3	−0.1	0.1	−0.5	−0.2	−0.0	−0.0	0.0	0.1
^{2,6}[MnCp$_2$]	−0.6	−0.2	0.1	−1.0	−0.4	−0.0	0.1	0.2	0.2
^{1,5}[FeL$_2$]	−0.3	−0.1	0.1	−0.5	−0.2	−0.0	0.1	0.1	0.1
^{3,5}[FeL$_2$] (1)	−0.3	−0.1	0.0	−0.4	−0.2	−0.0	0.1	0.1	0.1
^{3,5}[FeL$_2$] (2)	−0.3	−0.1	0.1	−0.4	−0.1	0.0	0.2	0.2	0.2
^{2,6}[FeL$_2$Cl]	−0.2	−0.0	0.1	−0.6	−0.2	0.1	0.7	0.5	0.3
^{4,6}[FeL$_2$Cl]	−0.2	−0.0	0.1	−0.4	−0.1	0.0	0.3	0.2	0.2
^{1,5}[FeL$_2$NH$_3$]	−0.4	−0.1	0.1	−0.6	−0.2	0.0	0.2	0.2	0.2
^{3,5}[FeL$_2$NH$_3$]	−0.3	−0.1	0.1	−0.4	−0.1	0.0	0.3	0.2	0.1
|Max| [g]	0.9	0.3	0.2	1.4	0.5	0.1	0.7	0.5	0.3
MSD [h]	−0.4	−0.1	0.1	−0.6	−0.2	0.0	0.1	0.2	0.2
MAD [i]	0.4	0.1	0.1	0.6	0.2	0.0	0.2	0.2	0.2

[a]BSIE values in kcal/mol, with respect to the corresponding reference CBS limits from Table 3, main article. [b]Only valence electrons correlated (TM 3s3p in the frozen core, FC). [c]This table contains numeric data corresponding to FC-triples-related boxplots shown in Figure 2, main article. [c](T) correction for conventional calculations. [d]Marchetti–Werner (T*) correction, eq. (8), main article. [e]CBS extrapolation using eq. (2), main article. [f]Schwenke-style CBS extrapolation, eq. (4), main article. [g]Maximum absolute deviation. [h]Mean signed deviation. [i]Mean absolute deviation.

Table S9: BSIE of the Δ3s3p Contribution to Spin–State Energetics. [a,b]

	CCSD(T)			CCSD(T)-F12a	CCSD(T)-F12b
	acT	acQ	ac[T:Q][c]	acT	acT
^{1,5}[Fe(NCH)$_6$]$^{2+}$	0.1	0.0	0.0	−0.0	−0.0
^{2,6}[Fe(NCH)$_6$]$^{3+}$	−0.5	−0.3	−0.2	−0.4	−0.2
^{2,4}[Co(NCH)$_6$]$^{2+}$	−0.0	−0.0	−0.0	−0.0	−0.0
^{1,5}[Fe(CNH)$_6$]$^{2+}$	0.2	0.1	−0.0	0.1	0.1
^{2,6}[Fe(CNH)$_6$]$^{3+}$	−0.4	−0.3	−0.2	−0.1	−0.0
^{3,5}[FeO(NH$_3$)$_5$]$^{2+}$	−0.1	−0.1	−0.1	−0.2	−0.0
^{1,5}[Fe(H$_2$O)$_6$]$^{2+}$	0.3	−0.1	−0.3	−0.2	−0.1
^{4,6}[Fe(H$_2$O)$_6$]$^{3+}$ (ve)	−0.2	−0.2	−0.2	−0.3	−0.2
^{1,3}[FeCp$_2$]	0.1	0.1	0.1	0.1	0.1
^{1,3}[FeCp$_2$] (ve)	0.2	0.1	−0.0	−0.0	0.0
^{2,6}[MnCp$_2$]	0.0	−0.0	−0.1	−0.3	−0.2
^{1,5}[FeL$_2$]	−0.1	−0.2	−0.2	−0.1	−0.1
^{3,5}[FeL$_2$] (1)	−0.1	−0.1	−0.1	−0.2	−0.1
^{3,5}[FeL$_2$] (2)	−0.1	−0.1	−0.1	−0.1	−0.1
^{2,6}[FeL$_2$Cl]	−0.5	−0.4	−0.2	−0.3	−0.2
^{4,6}[FeL$_2$Cl]	−0.3	−0.2	−0.2	−0.2	−0.1
^{1,5}[FeL$_2$NH$_3$]	−0.1	−0.1	−0.2	−0.1	−0.1
^{3,5}[FeL$_2$NH$_3$]	0.0	−0.0	−0.1	−0.0	−0.0
|Max| [d]	0.5	0.4	0.3	0.4	0.2
MSD [e]	−0.1	−0.1	−0.1	−0.1	−0.1
MAD [f]	0.2	0.1	0.1	0.2	0.1

[a]BSIE values in kcal/mol, with respect to the corresponding reference CBS limits from Table 3, main article. [b]This table contains numeric data corresponding to boxplots in Figure 3, main article. [c]Correlation energy extrapolated using eq. (2), reference energy taken from the larger basis set. [d]Maximum absolute deviation. [e]Mean signed deviation. [f]Mean absolute deviation.

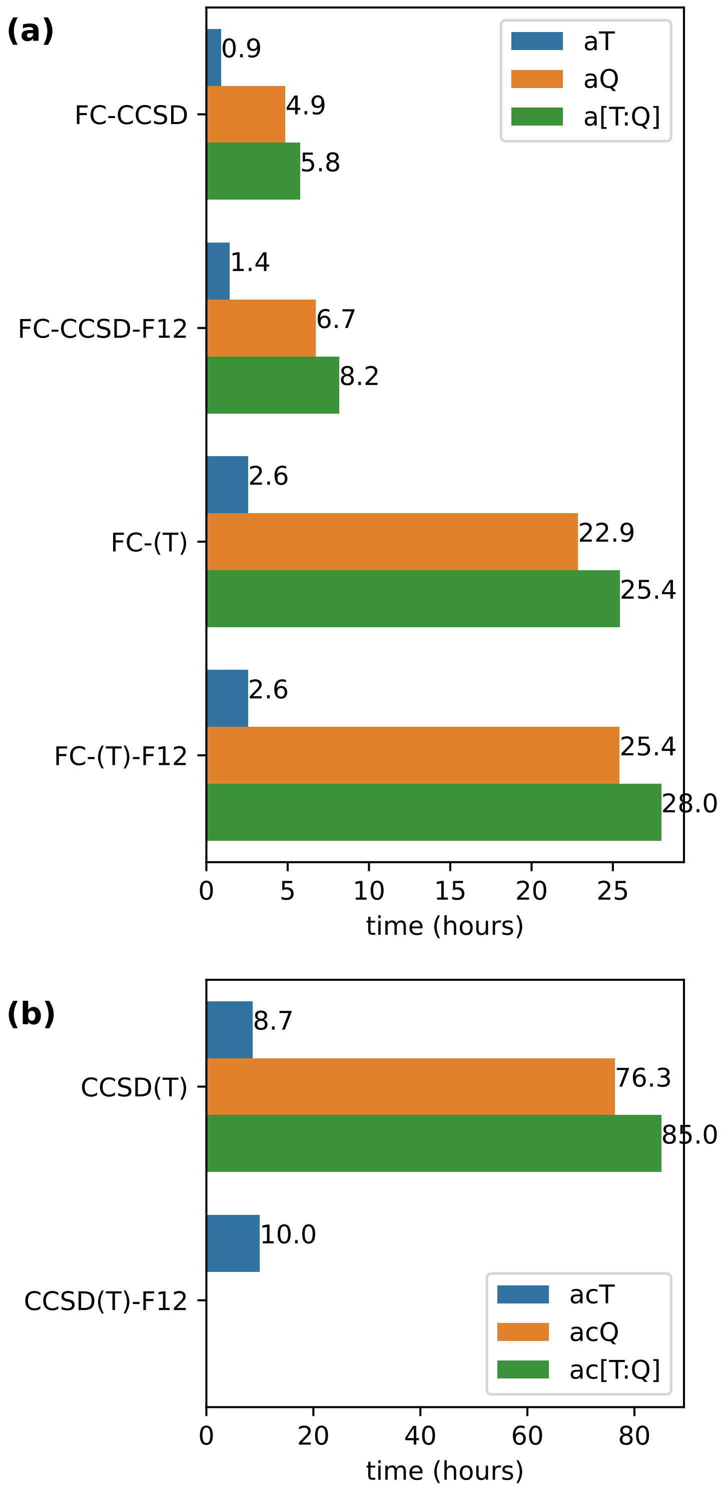

Figure S1: Wall-clock times of selected conventional and explicitly correlated CCSD(T) calculations of the quintet–singlet splitting for $[Fe(NCH)_6]^{2+}$: (a) FC-CCSD and FC-(T) terms analyzed in Figure 2, main article, and Tables S7, S8; (b) Δ3s3p correction analyzed in Figure 3, main article, and Table S9. Note that the timings for FC-(T) terms in part (a) do *not* include the preceding FC-CCSD calculations. For details of the time measurements, see caption of Figure 7, main article.

Table S10: BSIE of the FC-CCSD-F12a Spin–State Energetics Calculated Using Several Basis Sets of Triple-ζ or Lower Quality. [a,b]

	aT	T	cT	cT(D)	cT/D
^{1,5}[Fe(NCH)$_6$]$^{2+}$	−1.3	−1.4	0.1	0.1	1.4
^{2,6}[Fe(NCH)$_6$]$^{3+}$	−2.1	−2.2	0.1	0.3	1.9
^{2,4}[Co(NCH)$_6$]$^{2+}$	−0.8	−0.9	−0.0	0.0	0.6
^{1,5}[Fe(CNH)$_6$]$^{2+}$	−0.8	−1.0	0.4	−0.1	−0.4
^{2,6}[Fe(CNH)$_6$]$^{3+}$	−1.9	−2.1	0.1	−0.0	0.1
^{3,5}[FeO(NH$_3$)$_5$]$^{2+}$	−0.6	−0.6	0.2	0.5	1.8
^{1,5}[Fe(H$_2$O)$_6$]$^{2+}$	−1.1	−1.4	−0.1	−0.3	−0.9
^{4,6}[Fe(H$_2$O)$_6$]$^{3+}$ (ve)	−1.3	−1.2	0.3	0.3	0.9
^{1,3}[FeCp$_2$]	0.2	0.4	0.7	0.7	1.2
^{1,3}[FeCp$_2$] (ve)	0.2	0.5	0.7	0.7	1.1
^{2,6}[MnCp$_2$]	−1.0	−0.8	0.9	0.9	2.7
^{1,5}[FeL$_2$]	−1.2	−1.3	0.2	0.1	0.8
^{3,5}[FeL$_2$] (1)	−0.8	−0.7	0.3	0.6	1.5
^{3,5}[FeL$_2$] (2)	−0.7	−0.7	0.3	0.4	1.5
^{2,6}[FeL$_2$Cl]	−1.6	−1.7	0.6	0.9	3.0
^{4,6}[FeL$_2$Cl]	−1.3	−1.4	0.1	0.6	1.6
^{1,5}[FeL$_2$NH$_3$]	−1.1	−1.1	0.4	0.7	2.2
^{3,5}[FeL$_2$NH$_3$]	−0.6	−0.6	0.4	0.5	1.5
|Max| [c]	2.1	2.2	0.9	0.9	3.0
MSD [d]	−1.0	−1.0	0.3	0.4	1.3
MAD [e]	1.0	1.1	0.3	0.4	1.4

[a]BSIE values in kcal/mol, with respect to the corresponding reference CBS limits from Table 3, main article. [b]This table contains numeric data corresponding to boxplots in Figure 4, main article. [c]Maximum absolute deviation. [d]Mean signed deviation. [e]Mean absolute deviation.

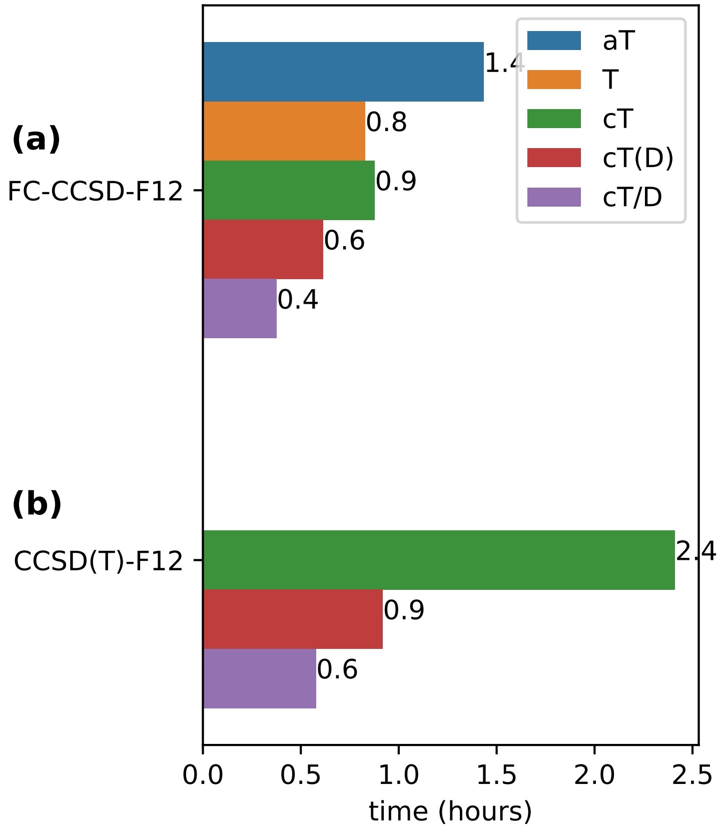

Figure S2: Wall-clock times for selected calculations of the quintet–singlet splitting for $[Fe(NCH)_6]^{2+}$: (a) FC-CCSD-F12 calculations using five different basis set corresponding to data shown in Figure 4, main article, and Table S10; (b) CCSD(T)-F12 (with valence plus 3s3p electrons correlated) using the three core-augmented basis sets. For details of the time measurements, see caption of Figure 7, main article.

Table S11: Effect of Diffuse Functions on Conventional FC-CCSD and Explicitly Correlated FC-CCSD-F12a Spin–State Energetics[a]

	FC-CCSD	FC-CCSD-F12a
^{1,5}[Fe(NCH)$_6$]$^{2+}$	2.2	0.1
^{2,6}[Fe(NCH)$_6$]$^{3+}$	1.1	0.1
^{2,4}[Co(NCH)$_6$]$^{2+}$	1.0	0.1
^{1,5}[Fe(CNH)$_6$]$^{2+}$	2.6	0.2
^{2,6}[Fe(CNH)$_6$]$^{3+}$	1.6	0.2
^{3,5}[FeO(NH$_3$)$_5$]$^{2+}$	0.7	0.0
^{1,5}[Fe(H$_2$O)$_6$]$^{2+}$	0.4	0.4
^{4,6}[Fe(H$_2$O)$_6$]$^{3+}$ (ve)	0.2	−0.0
^{1,3}[FeCp$_2$]	0.9	−0.3
^{1,3}[FeCp$_2$] (ve)	0.3	−0.2
^{2,6}[MnCp$_2$]	1.4	−0.1
^{1,5}[FeL$_2$]	1.2	0.1
^{3,5}[FeL$_2$] (1)	0.5	−0.1
^{3,5}[FeL$_2$] (2)	0.7	−0.0
^{2,6}[FeL$_2$Cl]	0.9	0.1
^{4,6}[FeL$_2$Cl]	0.4	0.1
^{1,5}[FeL$_2$NH$_3$]	1.3	0.0
^{3,5}[FeL$_2$NH$_3$]	0.6	−0.0
|Max| [b]	2.6	0.4
MAD [c]	1.0	0.1

[a]Obtained by comparison of relative energies calculated using aT and T basis sets, values in kcal/mol. [b]Maximum absolute deviation. [c]Mean absolute deviation.

Table S12: Comparison of the /T and /cT Energies Obtained Using Conventional and Explicitly-Correlated FC-CCSD Calculations

	^{1,5}[Fe(NCH)$_6$]$^{2+}$	^{2,6}[Fe(NCH)$_6$]$^{3+}$	^{1,5}Fe^{2+}	^{2,6}Fe^{3+}
FC-CCSD/T	−28.6	−42.4	−59.8	−132.8
FC-CCSD/cT	−27.3	−40.6	−59.1	−131.2
difference FC-CCSD	1.2	1.9	0.7	1.6
FC-CCSD-F12a/T	−24.1	−38.6		
FC-CCSD-F12a/cT	−22.5	−36.3		
difference FC-CCSD-F12a	1.6	2.3		
FC-CCSD-F12b/T	−25.4	−40.2		
FC-CCSD-F12b/cT	−23.4	−37.3		
difference FC-CCSD-F12b	2.0	2.9		

[a] Values in kcal/mol. [b] Fe$^{2+/3+}$ are bare ions surrounded by 6 point charges −0.5e in the octahedral arrangement at 1.131 Ådistance from the central Fe; d orbitals are occupied analogously as for the molecular complexes.

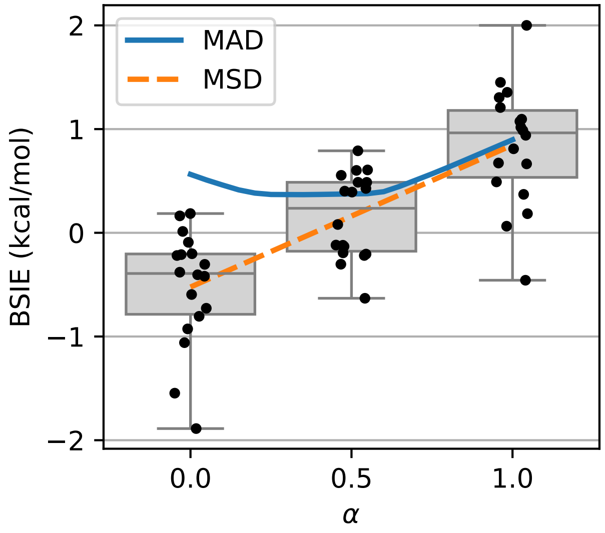

Figure S3: BSIE of the CCSD(T,α)-F12a/cT(D) spin–state energetics, where (Tα) is defined by eq. (10), main article, as a function of the α parameter. The mean absolute deviation (MAD) and mean signed deviation (MSD) are drawn as functions of the α parameter and statistical box-plots are shown for three representative values: $\alpha = 0$ corresponding to the unscaled (T) correction, $\alpha = 1$ corresponding to the Marchetti–Werner (T*) correction, and $\alpha = 1/2$ corresponding to the (T#) correction proposed in this work. The corresponding numeric data can be found in Table S15.

Table S13: BSIE for FC-(T), (T*), (T#) Terms in CCSD(T)-F12a/cT(D) Calculations. [a,b,c]

	FC-F12a(T)[d]	FC-F12a(T*)[e]	FC-F12a(T#)[f]
^{1,5}[Fe(NCH)$_6$]$^{2+}$	−1.0	0.5	−0.2
^{2,6}[Fe(NCH)$_6$]$^{3+}$	−0.7	0.7	0.0
^{2,4}[Co(NCH)$_6$]$^{2+}$	−0.5	0.1	−0.2
^{1,5}[Fe(CNH)$_6$]$^{2+}$	−1.6	1.5	−0.0
^{2,6}[Fe(CNH)$_6$]$^{3+}$	−1.1	1.2	0.0
^{3,5}[FeO(NH$_3$)$_5$]$^{2+}$	−0.5	−0.0	−0.2
^{1,5}[Fe(H$_2$O)$_6$]$^{2+}$	−0.4	−0.1	−0.3
^{4,6}[Fe(H$_2$O)$_6$]$^{3+}$ (ve)	−0.2	0.3	0.0
^{1,3}[FeCp$_2$]	−0.5	0.2	−0.2
^{1,3}[FeCp$_2$] (ve)	−0.4	0.2	−0.1
^{2,6}[MnCp$_2$]	−0.9	0.6	−0.1
^{1,5}[FeL$_2$]	−0.6	0.5	−0.1
^{3,5}[FeL$_2$] (1)	−0.3	0.7	0.2
^{3,5}[FeL$_2$] (2)	−0.5	0.7	0.1
^{2,6}[FeL$_2$Cl]	−0.8	1.6	0.4
^{4,6}[FeL$_2$Cl]	−0.5	0.7	0.1
^{1,5}[FeL$_2$NH$_3$]	−0.8	0.8	−0.0
^{3,5}[FeL$_2$NH$_3$]	−0.5	0.8	0.1
|Max| [g]	1.6	1.6	0.4
MSD [h]	−0.7	0.6	−0.0
MAD [i]	0.7	0.6	0.1

[a]BSIE values in kcal/mol, with respect to the corresponding reference CBS limits from Table 3, main article. [b]Only valence electron correlation (TM 3s3p in frozen core, FC). [c]These are numeric data for boxplots presented in Figure 5, main article. [d]Unscaled (T) based on CCSD-F12a amplitudes. [e]Marchetti–Werner scaling, eq. (8), main article. [f](Tα) scaling proposed in this work, eq. (10), main article, with $\alpha = 1/2$. [g]Maximum absolute deviation. [h]Mean signed deviation. [i]Mean absolute deviation.

Table S14: BSIE of the Δ3s3p Contribution to Spin–State Energetics at the CCSD(T)-F12a/cT(D) Level With Different Formulations of the (T) Term and Different Values of the Geminal Exponent γ. [a]

	(T)[b] $\gamma = 1.0$	(T*)-F12a [c] $\gamma = 1.0$	(T#)-F12a [d] $\gamma = 1.0$	(T#)-F12a [d] $\gamma = 1.4$
$^{1,5}[Fe(NCH)_6]^{2+}$	−0.2	−0.0	−0.1	0.0
$^{2,6}[Fe(NCH)_6]^{3+}$	−0.5	−0.4	−0.4	−0.4
$^{2,4}[Co(NCH)_6]^{2+}$	−0.1	0.0	−0.1	0.0
$^{1,5}[Fe(CNH)_6]^{2+}$	−0.2	0.0	−0.1	0.1
$^{2,6}[Fe(CNH)_6]^{3+}$	−0.4	−0.2	−0.3	−0.2
$^{3,5}[FeO(NH_3)_5]^{2+}$	−0.3	−0.4	−0.3	−0.3
$^{1,5}[Fe(H_2O)_6]^{2+}$	−0.0	0.0	−0.0	−0.1
$^{4,6}[Fe(H_2O)_6]^{3+}$ (ve)	−0.3	−0.2	−0.3	−0.3
$^{1,3}[FeCp_2]$	0.0	0.1	0.1	0.1
$^{1,3}[FeCp_2]$ (ve)	−0.0	−0.0	−0.0	−0.0
$^{2,6}[MnCp_2]$	−0.4	−0.3	−0.4	−0.2
$^{1,5}[FeL_2]$	−0.2	−0.1	−0.1	−0.1
$^{3,5}[FeL_2]$ (1)	−0.3	−0.2	−0.2	−0.2
$^{3,5}[FeL_2]$ (2)	−0.1	−0.1	−0.1	−0.1
$^{2,6}[FeL_2Cl]$	−0.5	−0.6	−0.6	−0.4
$^{4,6}[FeL_2Cl]$	−0.4	−0.3	−0.3	−0.3
$^{1,5}[FeL_2NH_3]$	−0.2	−0.1	−0.2	−0.1
$^{3,5}[FeL_2NH_3]$	−0.1	−0.0	−0.0	0.0
|Max| [e]	0.5	0.6	0.6	0.4
MSD [f]	−0.2	−0.2	−0.2	−0.1
MAD [g]	0.2	0.2	0.2	0.2

[a]BSIE values in kcal/mol, with respect to the corresponding reference CBS limits from Table 3, main article. [b]Unscaled (T) based on CCSD-F12a amplitudes. [c]Marchetti–Werner scaling, eq. (8), main article. [d](Tα) scaling proposed in this work, eq. (10), main article, with $\alpha = 1/2$. [e]Maximum absolute deviation. [f]Mean signed deviation. [g]Mean absolute deviation.

Table S15: BSIE of the CCSD(T)-F12a/cT(D) Spin–State Energetics with Different Variants of the (T) Term. [a,b]

	CCSD(T)-F12a [c]	CCSD(T*)-F12a [d]	CCSD(T#)-F12a [e]
^{1,5}[Fe(NCH)$_6$]$^{2+}$	−1.1	0.7	−0.2
^{2,6}[Fe(NCH)$_6$]$^{3+}$	−0.9	0.7	−0.1
^{2,4}[Co(NCH)$_6$]$^{2+}$	−0.6	0.2	−0.2
^{1,5}[Fe(CNH)$_6$]$^{2+}$	−1.9	1.5	−0.2
^{2,6}[Fe(CNH)$_6$]$^{3+}$	−1.5	0.9	−0.3
^{3,5}[FeO(NH$_3$)$_5$]$^{2+}$	−0.3	0.1	−0.1
^{1,5}[Fe(H$_2$O)$_6$]$^{2+}$	−0.8	−0.5	−0.6
^{4,6}[Fe(H$_2$O)$_6$]$^{3+}$ (ve)	−0.2	0.4	0.1
^{1,3}[FeCp$_2$]	0.2	1.0	0.6
^{1,3}[FeCp$_2$] (ve)	0.2	0.8	0.5
^{2,6}[MnCp$_2$]	−0.4	1.2	0.4
^{1,5}[FeL$_2$]	−0.7	0.5	−0.1
^{3,5}[FeL$_2$] (1)	0.0	1.1	0.6
^{3,5}[FeL$_2$] (2)	−0.2	1.1	0.4
^{2,6}[FeL$_2$Cl]	−0.4	2.0	0.8
^{4,6}[FeL$_2$Cl]	−0.2	1.0	0.4
^{1,5}[FeL$_2$NH$_3$]	−0.4	1.4	0.5
^{3,5}[FeL$_2$NH$_3$]	−0.1	1.3	0.6
|Max| [f]	1.9	2.0	0.8
MSD [g]	−0.5	0.8	0.2
MAD [h]	0.6	0.9	0.4

[a]BSIE values in kcal/mol, with respect to the corresponding reference CBS limits from Table 3, main article. [b]These are numeric values corresponding to boxplots show in Figure S15, Supporting Information. [c]Unscaled (T) based on CCSD-F12a amplitudes. [d]Marchetti–Werner scaling, eq. (8), main article. [e](Tα) scaling proposed in this work, eq. (10), main article, with $\alpha = 1/2$. [f]Maximum absolute deviation. [g]Mean signed deviation. [h]Mean absolute deviation.

5 Results of Other Methods, BSIE Transferability

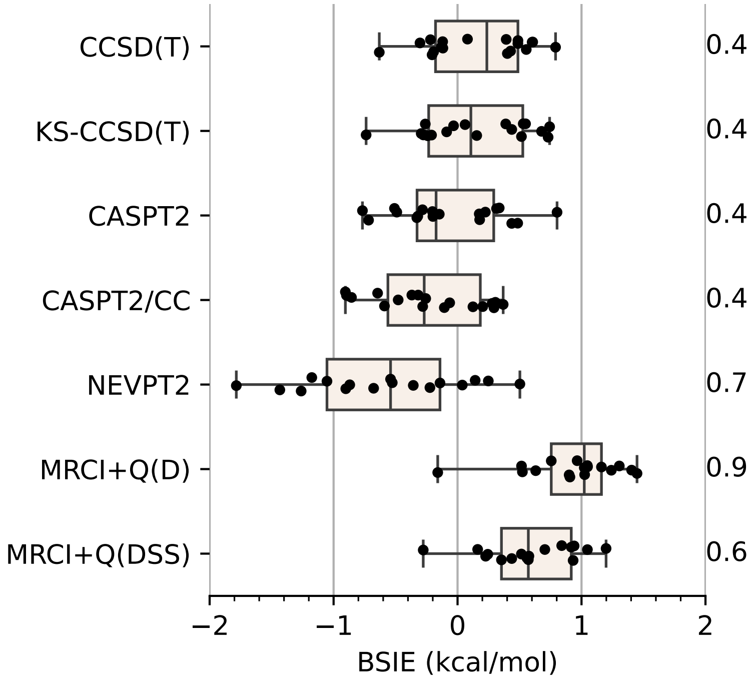

Figure S4: BSIEs for approximate CBS limits of different methods estimated using additive approximation of eq. (16), main article. Annotated values are mean absolute deviations (MADs).

Table S16: Spin–State Energetics from Various WFT Methods in Their CBS Limits. [a,b]

	KS-CCSD(T)[c]	MP2	CASPT2	CASPT2/CC	NEVPT2	MRCI+Q(D)[d]	MRCI+Q(DSS)[e]
$^{1,5}[Fe(NCH)_6]^{2+}$	−3.8	−9.3	−9.3	−4.0	−14.8	−18.2	−12.7
$^{2,6}[Fe(NCH)_6]^{3+}$	−17.2	−26.2	−22.2	−16.9	−20.7	−27.5	−22.7
$^{2,4}[Co(NCH)_6]^{2+}$	−9.7	−12.8	−16.8	−13.4	−21.4	−19.7	−16.9
$^{1,5}[Fe(CNH)_6]^{2+}$	53.8	60.1	61.8	63.2	65.1	34.8	43.2
$^{2,6}[Fe(CNH)_6]^{3+}$	30.2	32.3	34.5	36.6	27.5	17.2	23.6
$^{3,5}[FeO(NH_3)_5]^{2+}$	2.3	6.3	−3.3	−0.3	*f*	*f*	*f*
$^{1,5}[Fe(H_2O)_6]^{2+}$	−43.4	−52.3	−54.3	−46.1	−35.8	−43.1	−42.2
$^{4,6}[Fe(H_2O)_6]^{3+}$ (ve)	−47.9	−56.5	−51.8	−50.5	−43.2	−51.8	−49.6
$^{1,3}[FeCp_2]$	35.6	52.4	40.4	39.6	48.9	31.4	33.0
$^{1,3}[FeCp_2]$ (ve)	49.3	55.4	47.8	48.4	55.7	47.2	47.6
$^{2,6}[MnCp_2]$	2.0	−0.6	4.5	6.0	16.2	−5.7	−1.3
$^{1,5}[FeL_2]$	−29.6	−38.9	−38.1	−32.2	−27.0	−34.3	−31.6
$^{3,5}[FeL_2]$ (1)	5.4	−0.2	0.9	3.6	3.4	−1.1	1.5
$^{3,5}[FeL_2]$ (2)	8.9	1.2	3.3	5.6	3.3	−0.8	2.4
$^{2,6}[FeL_2Cl]$	−5.4	−15.1	−9.5	−5.6	−2.2	−16.2	−11.9
$^{4,6}[FeL_2Cl]$	−0.9	−6.0	−2.4	−0.6	4.2	−8.7	−4.9
$^{1,5}[FeL_2NH_3]$	−9.1	−19.2	−17.6	−11.7	−6.3	−20.6	−15.8
$^{3,5}[FeL_2NH_3]$	3.3	−4.9	−1.7	0.3	−0.7	−7.6	−3.8

[a]Values in kcal/mol. [b]CBS extrapolation ac[Q:5]/Q using eq. (2) for correlation energy and eq. (3)for reference energy. [c]Using B3LYP orbitals. [d]With Davidson correction. [e]With Davidson–Silver–Siegbahn correction. [f]Calculations not performed.

Table S17: Spin–State Energetics from Various WFT Methods Using cT(D) Basis Set. [a]

	KS-CCSD(T)[b]	MP2	CASPT2	CASPT2/CC	NEVPT2	MRCI+Q(D)[c]	MRCI+Q(DSS)[d]
$^{1,5}[Fe(NCH)_6]^{2+}$	−9.0	−14.8	−14.5	−9.6	−20.8	−22.1	−17.2
$^{2,6}[Fe(NCH)_6]^{3+}$	−22.0	−31.6	−27.4	−22.5	−26.4	−31.3	−26.9
$^{2,4}[Co(NCH)_6]^{2+}$	−12.4	−15.5	−19.4	−16.2	−24.4	−21.7	−19.1
$^{1,5}[Fe(CNH)_6]^{2+}$	47.5	53.4	54.9	56.2	57.2	30.1	37.3
$^{2,6}[Fe(CNH)_6]^{3+}$	24.4	26.0	28.2	30.2	20.6	12.7	18.2
$^{3,5}[FeO(NH_3)_5]^{2+}$	−0.1	3.6	−6.0	−3.0	[e]	[e]	[e]
$^{1,5}[Fe(H_2O)_6]^{2+}$	−45.8	−54.9	−56.4	−48.4	−38.5	−45.0	−44.2
$^{4,6}[Fe(H_2O)_6]^{3+}$ (ve)	−49.8	−58.6	−53.0	−52.3	−45.4	−53.2	−51.1
$^{1,3}[FeCp_2]$	33.6	49.6	38.0	37.1	46.4	29.3	30.8
$^{1,3}[FeCp_2]$ (ve)	48.5	54.0	46.5	47.2	54.7	46.2	46.5
$^{2,6}[MnCp_2]$	−3.2	−6.6	−1.4	0.0	9.7	−10.3	−6.3
$^{1,5}[FeL_2]$	−34.7	−43.8	−42.8	−37.1	−32.3	−38.3	−35.9
$^{3,5}[FeL_2]$ (1)	3.0	−2.8	−1.6	0.9	0.6	−3.1	−0.6
$^{3,5}[FeL_2]$ (2)	5.8	−1.9	0.2	2.5	−0.1	−3.1	−0.2
$^{2,6}[FeL_2Cl]$	−9.8	−20.3	−15.1	−11.2	−8.6	−19.8	−15.8
$^{4,6}[FeL_2Cl]$	−1.8	−7.3	−3.9	−2.1	2.4	−8.9	−5.3
$^{1,5}[FeL_2NH_3]$	−13.5	−23.6	−21.9	−16.4	−11.5	−23.8	−19.4
$^{3,5}[FeL_2NH_3]$	0.8	−7.7	−4.3	−2.5	−3.8	−9.4	−5.8

[a]Values in kcal/mol. [b]Using B3LYP orbitals. [c]With Davidson correction. [d]With Davidson–Silver–Siegbahn correction. [e]Calculations not performed.

Table S18: Differential BSIEs With Respect to CCSD(T) Using cT(D) Basis Set.[a]

	KS-CCSD(T)[a]	MP2	CASPT2	CASPT2/CC	NEVPT2	MRCI+Q(D)[c]	MRCI+Q(DSS)[d]
$^{1,5}[Fe(NCH)_6]^{2+}$	−0.1	−0.4	−0.1	−0.5	−1.0	1.2	0.6
$^{2,6}[Fe(NCH)_6]^{3+}$	0.0	−0.5	−0.4	−0.7	−0.8	1.0	0.7
$^{2,4}[Co(NCH)_6]^{2+}$	−0.1	0.0	0.1	−0.1	−0.3	0.7	0.4
$^{1,5}[Fe(CNH)_6]^{2+}$	0.0	−0.3	−0.5	−0.7	−1.6	1.6	0.4
$^{2,6}[Fe(CNH)_6]^{3+}$	0.0	−0.5	−0.5	−0.6	−1.1	1.3	0.5
$^{3,5}[FeO(NH_3)_5]^{2+}$	0.1	−0.2	−0.2	−0.2	[e]	[e]	[e]
$^{1,5}[Fe(H_2O)_6]^{2+}$	−0.1	−0.3	0.3	0.0	−0.4	0.5	0.4
$^{4,6}[Fe(H_2O)_6]^{3+}$ (ve)	0.0	−0.2	0.7	0.0	−0.3	0.6	0.4
$^{1,3}[FeCp_2]$	0.1	−0.5	−0.3	−0.2	−0.4	0.2	0.0
$^{1,3}[FeCp_2]$ (ve)	0.2	−0.3	−0.3	−0.2	0.0	0.0	−0.1
$^{2,6}[MnCp_2]$	0.1	−0.8	−0.6	−0.8	−1.3	0.6	0.3
$^{1,5}[FeL_2]$	−0.1	0.1	0.3	0.1	−0.2	1.0	0.7
$^{3,5}[FeL_2]$ (1)	0.0	−0.2	−0.1	−0.3	−0.4	0.5	0.3
$^{3,5}[FeL_2]$ (2)	0.0	0.0	0.0	−0.1	−0.4	0.7	0.5
$^{2,6}[FeL_2Cl]$	0.0	−0.9	−1.3	−1.3	−2.1	0.7	0.4
$^{4,6}[FeL_2Cl]$	0.0	−0.5	−0.6	−0.6	−0.9	0.6	0.5
$^{1,5}[FeL_2NH_3]$	−0.3	−0.3	−0.3	−0.6	−1.2	0.8	0.5
$^{3,5}[FeL_2NH_3]$	−0.1	−0.5	−0.3	−0.4	−0.7	0.6	0.4
|Max|	0.3	0.9	1.3	1.3	2.1	1.6	0.7
MSD	0.0	−0.4	−0.2	−0.4	−0.8	0.7	0.4
MAD	0.1	0.4	0.4	0.4	0.8	0.7	0.4

[a]Values in kcal/mol. These are numeric data for the box-plot in Figure 8(b), main article. [b]Using B3LYP orbitals. [c]With Davidson correction. [d]With Davidson–Silver–Siegbahn correction. [e]Calculations not performed.

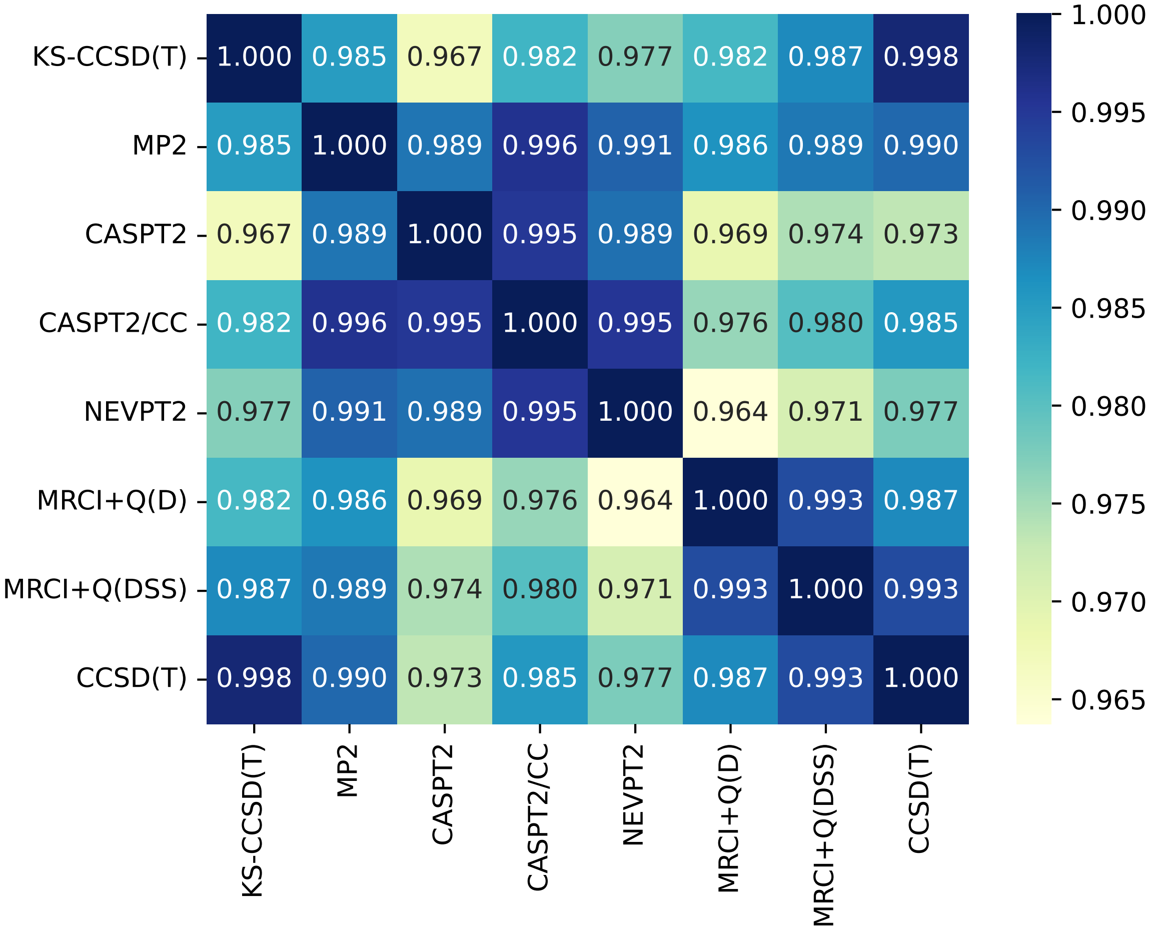

Figure S5: Heat-map of the Pearson's correlation matrix between the BSIEs of all considered WFT methods calculated for the benchmark set of spin–state energetics using the cT(D) basis set.

6 Additional Calculations for $MnCp_2$

In the additional calculations of the doublet–sextet energy difference for $MnCp_2$, whose results are presented in Figure 10, main article, we used the set of geometries from Drousou et al.[S18] In order to reduce the computational cost of canonical CCSD(T) calculations, these geometries were perfected to C_{2h} symmetry, which was estimated to influence the relative energy by no more than 0.1 kcal/mol (Table S19).

The scalar-relativistic corrections at DK level (ΔDK) were added to non-relativistic estimates of the CCSD(T)/CBS energies. Although the results in ref S18 were computed with the ZORA Hamiltonian, the difference to the presently chosen DK Hamiltonian is expected to alter the relative energy difference by no more than 0.3 kcal/mol (Table S19). The relativistic correction for $MnCp_2$ doublet–sextet splitting is quite significant, 2.6–2.9 kcal/mol, but well transferable between different methods.

Another small difference is that the present CCSD(T) calculations in Molpro are based on the ROHF-type orbitals, whereas the DPLNO-CCSD(T) ones from ref S18 are based on quasi-restricted orbitals obtained from earlier UHF-type calculations (QRO–UHF). This is not expected to yield major difference in the relative energies and test calculations indeed show that DLPNO-CCSD(T) energies for both choices of the reference orbitals (ROHF, QRO–UHF) are identical to within 0.3 kcal/mol (Table S20).

Additional estimates of the CBS limits indicated in Figure 10, main article, as "CBS + ΔDK" were obtained (after adding the ΔDK correction described above) to the results of CCSD(T#)-F12a/cT(D) calculations in the case of calculations with HF reference orbitals. The results for KS reference orbitals were obtained assuming the BSIE transferability [eq. (16), main article].

Table S19: Comparison of Scalar-Relativistic (ZORA, DK) and Nonrelativistic (NR) Results for Different Structures of $MnCp_2$. [a,b,c]

	C_{2h} [d]	original [d]
B3LYP-NR	−6.3	−6.2
B3LYP-ZORA	−3.3	−3.3
B3LYP-DK	−3.7	−3.7
Δ(ZORA)-B3LYP	2.9	2.9
Δ(DK)-B3LYP	2.6	2.6
CCSD(T)-NR	−6.2	NA
CCSD(T)-DK	−3.3	NA
Δ(DK)-CCSD(T)	2.9	NA

[a]Doublet–sextet splitting, values in kcal/mol. [b]B3LYP-NR using def2-TZVP, B3LYP-ZORA using ZORA-def2-TZVP, B3LYP-DK using DK-def2-TZVP basis sets. [c]CCSD(T)-NR using cT(D) basis set, CCSD(T)-DK using cT(D)-DK basis set. [d]Original structures from S18 or symmetrized to C_{2h} point group.

Table S20: Comparison of DLPNO-CCSD(T0) Results for Doublet–Sextet Splitting of $MnCp_2$ Using Different Reference Orbitals. [a,b,c]

	ΔE_{ref}	ΔE_{CCSD}	$\Delta E_{\mathrm{CCSD(T)}}$
ROHF	−111.3	−35.7	−27.5
QRO–UHF	−111.3	−35.9	−27.8

[a]Values in kcal/mol. [b]Basis set: def2-TZVP for Mn, C; def2-SVP for H. [c]NormalPNO settings.

7 Sample Input file for CCSD(T#)-F12a Calculations

Sample Molpro input file is provided below for the quintet state of $[Fe(H_2O)_6]^{2+}$

```
***[Fe(H2O)6]2+,quint
print,basis,orbitals
memory,300,m
file,2,wfu,old
symmetry,X,Y,Z,noorient
geometry={
19
geometry from Wibraham et al. 2017
Fe  0.000000 0.000000 0.000000
O  2.032306 -0.000000 -0.000000
O  0.000000 0.000000 2.005017
O  0.000000 2.033621 0.000000
O  -2.032306 0.000000 -0.000000
O  0.000000 0.000000 -2.005017
O  -0.000000 -2.033621 0.000000
H  2.619309 0.772765 0.000000
H  2.619309 -0.772765 -0.000000
H  -0.781928 0.000000 2.577493
H  0.781928 -0.000000 2.577493
H  -0.769817 2.622599 0.000000
H  0.769817 2.622599 0.000000
H  -2.619309 0.772765 -0.000000
H  -2.619309 -0.772765 0.000000
H  -0.781928 0.000000 -2.577493
H  0.781928 0.000000 -2.577493
H  -0.769817 -2.622599 -0.000000
H  0.769817 -2.622599 0.000000
}

basis={
Fe=cc-pwCVTZ
O=cc-pVTZ
H=cc-pVDZ
set,df
Fe=aug-cc-pVTZ/mp2fit
O=aug-cc-pVTZ/mp2fit
H=aug-cc-pVDZ/mp2fit
set,jk
Fe=def2-TZVPP/jkfit
O=aug-cc-pVTZ/jkfit
H=aug-cc-pVDZ/jkfit
set,ri
Fe=def2-TZVPP/jkfit
O=aug-cc-pVTZ/optri
H=aug-cc-pVDZ/optri
}

{hf
wf,charge=2,symmetry=4,spin=4
occ,14,7,7,3,7,3,3,0
closed,12,7,7,3,7,2,2,0
start,2101.2
save,2102.2
}

put,molden,molden.out

{uccsd(t)-f12,scale_trip=1,df_basis=df,df_basis_exch=jk,ri_basis=ri,shifts=0.6,shiftp=0.6,maxit=100
core,5,2,2,0,2,0,0,0
}

---
```

The input file is thus identical with that for CCSD(T*)-F12a calculations (mind the usage of option `scale_trip=1`), but non-standard processing of the output file is required in order to obtain the CCSD(T#)-F12a energy. Relevant results from the output file are:

```
 Ecorr(MP2)                            -2.148269763094
 Ecorr(MP2-F12)                        -2.442021684631
 Scale factor for triples energy        1.136738842851
 Reference energy                   -1718.406023603237
 CABS relaxation correction to RHF     -0.043782698575
 New reference energy               -1718.449806301812
 UCCSD-F12a correlation energy         -2.419579265614
 Triples (T) contribution (scaled)     -0.068706925871
!RHF-UCCSD(T)-F12a energy           -1720.938092493297
```

Note that the CABS-corrected reference energy is the one called "`New reference energy`." The quantity reported in the output file as "`Triples (T) contribution (scaled)`" is the (T*) term defined in eq. (8) of the main article:

$$\Delta E_{(\mathrm{T}^*)} = f \Delta E_{(\mathrm{T})}, \tag{S.9}$$

where $f = 1.136738842851$ is the scale factor (also reported above). The (T#) energy can be computed as the arithmetic mean of the (T*) energy and the unscaled (T) energy (see eq. (10), main article, with $\alpha = 0.5$). Table S21 summarizes the relevant results and explains how the CCSD(T#)-F12a energy is calculated.

Table S21: Results of Sample CCSD(T#)-F12a Calculations for $^5[Fe(H_2O)_6]^{2+}$.

quantity	value[a]	comment
E_{ref}	-1718.44980630	output file: `New reference energy`
$E_{\text{CCSD-F12a, corr}}$	-2.41957927	output file: `UCCSD-F12a correlation energy`
$E_{\text{CCSD-F12a}}$	-1720.86938557	calculated: $E_{\mathrm{ref}} + E_{\text{CCSD-F12a, corr}}$
$\Delta E_{(\mathrm{T}^*)}$	-0.06870693	output file: `Triples (T) contribution (scaled)`
$f = E_{\mathrm{corr}}^{\text{MP2-F12}} / E_{\mathrm{corr}}^{\text{MP2}}$	1.13673884	output file: `Scale factor for triples energy`
$\Delta E_{(\mathrm{T})}$	-0.06044214	calculated: $\Delta E_{(\mathrm{T}^*)}/f$
$\Delta E_{(\mathrm{T\#})}$	-0.06457453	calculated: $(\Delta E_{(\mathrm{T}^*)} + \Delta E_{(\mathrm{T})})/2$
$E_{\text{CCSD(T\#)-F12a}}$	-1720.93396010	calculated: $E_{\text{CCSD-F12a}} + \Delta E_{(\mathrm{T\#})}$

[a] All energies in atomic units.

8 Complete References 67, 69, and 76

Complere references 67, 69, and 76 (main article) are given below as refs S1, S2, and S3.

References

(S1) Werner, H.-J.; Knowles, P. J.; Celani, P.; Györffy, W.; Hesselmann, A.; Kats, D.; Knizia, G.; Köhn, A.; Korona, T.; Kreplin, D.; Lindh, R.; Ma, Q.; Manby, F. R.; Mitrushenkov, A.; Rauhut, G.; Schütz, M.; Shamasundar, K. R.; Adler, T. B.; Amos, R. D.; Bennie, S. J.; Bernhardsson, A.; Berning, A.; Black, J. A.; Bygrave, P. J.; Cimiraglia, R.; Cooper, D. L.; Coughtrie, D.; Deegan, M. J. O.; Dobbyn, A. J.; Doll, K.; Dornbach, M.; Eckert, F.; Erfort, S.; Goll, E.; Hampel, C.; Hetzer, G.; Hill, J. G.; Hodges, M.; Hrenar, T.; Jansen, G.; Köppl, C.; Kollmar, C.; Lee, S. J. R.; Liu, Y.; Lloyd, A. W.; Mata, R. A.; May, A. J.; Mussard, B.; McNicholas, S. J.; Meyer, W.; Miller III, T. F.; Mura, M. E.; Nicklass, A.; O'Neill, D. P.; Palmieri, P.; Peng, D.; Peterson, K. A.; Pflüger, K.; Pitzer, R.; Polyak, I.; Reiher, M.; Richardson, J. O.; Robinson, J. B.; Schröder, B.; Schwilk, M.; Shiozaki, T.; Sibaev, M.; Stoll, H.; Stone, A. J.; Tarroni, R.; Thorsteinsson, T.; Toulouse, J.; Wang, M.; Welborn, M.; Ziegler, B. MOLPRO, versions 2015–2022, a package of ab initio programs. see `http://www.molpro.net`.

(S2) Werner, H.-J.; Knowles, P. J.; Manby, F. R.; Black, J. A.; Doll, K.; Heßelmann, A.; Kats, D.; Köhn, A.; Korona, T.; Kreplin, D. A.; Ma, Q.; Miller, T. F.; Mitrushchenkov, A.; Peterson, K. A.; Polyak, I.; Rauhut, G.; Sibaev, M. The Molpro quantum chemistry package. *J. Chem. Phys.* **2020**, *152*, 144107.

(S3) Li Manni, G.; Fdez. Galván, I.; Alavi, A.; Aleotti, F.; Aquilante, F.; Autschbach, J.; Avagliano, D.; Baiardi, A.; Bao, J. J.; Battaglia, S.; Birnoschi, L.; Blanco-González, A.; Bokarev, S. I.; Broer, R.; Cacciari, R.; Calio, P. B.; Carlson, R. K.; Carvalho Couto, R.; Cerdán, L.; Chibotaru, L. F.; Chilton, N. F.; Church, J. R.; Conti, I.; Coriani, S.; Cuéllar-

Zuquin, J.; Daoud, R. E.; Dattani, N.; Decleva, P.; de Graaf, C.; Delcey, M. G.; De Vico, L.; Dobrautz, W.; Dong, S. S.; Feng, R.; Ferré, N.; Filatov(Gulak), M.; Gagliardi, L.; Garavelli, M.; González, L.; Guan, Y.; Guo, M.; Hennefarth, M. R.; Hermes, M. R.; Hoyer, C. E.; Huix-Rotllant, M.; Jaiswal, V. K.; Kaiser, A.; Kaliakin, D. S.; Khamesian, M.; King, D. S.; Kochetov, V.; Krośnicki, M.; Kumaar, A. A.; Larsson, E. D.; Lehtola, S.; Lepetit, M.-B.; Lischka, H.; López Ríos, P.; Lundberg, M.; Ma, D.; Mai, S.; Marquetand, P.; Merritt, I. C. D.; Montorsi, F.; Mörchen, M.; Nenov, A.; Nguyen, V. H. A.; Nishimoto, Y.; Oakley, M. S.; Olivucci, M.; Oppel, M.; Padula, D.; Pandharkar, R.; Phung, Q. M.; Plasser, F.; Raggi, G.; Rebolini, E.; Reiher, M.; Rivalta, I.; Roca-Sanjuán, D.; Romig, T.; Safari, A. A.; Sánchez-Mansilla, A.; Sand, A. M.; Schapiro, I.; Scott, T. R.; Segarra-Martí, J.; Segatta, F.; Sergentu, D.-C.; Sharma, P.; Shepard, R.; Shu, Y.; Staab, J. K.; Straatsma, T. P.; Sørensen, L. K.; Tenorio, B. N. C.; Truhlar, D. G.; Ungur, L.; Vacher, M.; Veryazov, V.; Voß, T. A.; Weser, O.; Wu, D.; Yang, X.; Yarkony, D.; Zhou, C.; Zobel, J. P.; Lindh, R. The OpenMolcas Web: A Community-Driven Approach to Advancing Computational Chemistry. *J. Chem. Theory Comput.* **2023**,

(S4) Pierloot, K.; Phung, Q. M.; Domingo, A. Spin State Energetics in First-Row Transition Metal Complexes: Contribution of (3s3p) Correlation and its Description by Second-Order Perturbation Theory. *J. Chem. Theory Comput.* **2017**, *13*, 537–553.

(S5) Hill, J. G.; Platts, J. A. Auxiliary basis sets for density fitting–MP2 calculations: Nonrelativistic triple-ζ all-electron correlation consistent basis sets for the 3d elements Sc–Zn. *J. Chem. Phys.* **2008**, *128*, 044104.

(S6) Bross, D. H.; Hill, J. G.; Werner, H.-J.; Peterson, K. A. Explicitly correlated composite thermochemistry of transition metal species. *J. Chem. Phys.* **2013**, *139*, 094302.

(S7) Weigend, F.; Köhn, A.; Hättig, C. Efficient use of the correlation consistent basis sets in resolution of the identity MP2 calculations. *J. Chem. Phys.* **2002**, *116*, 3175–3183.

(S8) Weigend, F. Hartree–Fock exchange fitting basis sets for H to Rn †. *J. Comp. Chem.* **2008**, *29*, 167–175.

(S9) Weigend, F. A fully direct RI-HF algorithm: Implementation, optimised auxiliary basis sets, demonstration of accuracy and efficiency. *Phys. Chem. Chem. Phys.* **2002**, *4*, 4285–4291.

(S10) Yousaf, K. E.; Peterson, K. A. Optimized complementary auxiliary basis sets for explicitly correlated methods: aug-cc-pVnZ orbital basis sets. *Chem. Phys. Lett.* **2009**, *476*, 303–307.

(S11) Schwenke, D. W. The extrapolation of one-electron basis sets in electronic structure calculations: How it should work and how it can be made to work. *J. Chem. Phys.* **2005**, *122*, 014107.

(S12) Truhlar, D. G. Basis-set extrapolation. *Chem. Phys. Lett.* **1998**, *294*, 45–48.

(S13) Klopper, W.; Kutzelnigg, W. Gaussian basis sets and the nuclear cusp problem. *Journal of Molecular Structure: THEOCHEM* **1986**, *135*, 339–356.

(S14) Karton, A.; Martin, J. M. L. Comment on: "Estimating the Hartree-Fock limit from finite basis set calculations" [Jensen F (2005) Theor Chem Acc 113:267]. *Theoretical Chemistry Accounts* **2006**, *115*, 330–333.

(S15) Neese, F.; Valeev, E. F. Revisiting the Atomic Natural Orbital Approach for Basis Sets: Robust Systematic Basis Sets for Explicitly Correlated and Conventional Correlated ab initio Methods? *J. Chem. Theory Comput.* **2011**, *7*, 33–43.

(S16) Kesharwani, M. K.; Sylvetsky, N.; Köhn, A.; Tew, D. P.; Martin, J. M. L. Do CCSD and approximate CCSD-F12 variants converge to the same basis set limits? The case of atomization energies. *J. Chem. Phys.* **2018**, *149*, 154109.

(S17) Hill, J. G.; Peterson, K. A.; Knizia, G.; Werner, H.-J. Extrapolating MP2 and CCSD explicitly correlated correlation energies to the complete basis set limit with first and second row correlation consistent basis sets. *J. Chem. Phys.* **2009**, *131*, 194105.

(S18) Drosou, M.; Mitsopoulou, C. A.; Pantazis, D. A. Spin-State Energetics of Manganese Spin Crossover Complexes: Comparison of Single-Reference and Multi-Reference Ab Initio Approaches. *Polyhedron* **2021**, 115399.
